# Caloric restriction delays yeast chronological aging by remodeling carbohydrate and lipid metabolism, altering peroxisomal and mitochondrial functionalities, and postponing the onsets of apoptotic and liponecrotic modes of regulated cell death

**DOI:** 10.18632/oncotarget.24604

**Published:** 2018-03-05

**Authors:** Anthony Arlia-Ciommo, Anna Leonov, Adam Beach, Vincent R. Richard, Simon D. Bourque, Michelle T. Burstein, Pavlo Kyryakov, Alejandra Gomez-Perez, Olivia Koupaki, Rachel Feldman, Vladimir I. Titorenko

**Affiliations:** ^1^ Department of Biology, Concordia University, Montreal, Quebec, Canada

**Keywords:** yeast, cellular aging, caloric restriction, metabolism, mitochondria

## Abstract

A dietary regimen of caloric restriction delays aging in evolutionarily distant eukaryotes, including the budding yeast *Saccharomyces cerevisiae*. Here, we assessed how caloric restriction influences morphological, biochemical and cell biological properties of chronologically aging yeast advancing through different stages of the aging process. Our findings revealed that this low-calorie diet slows yeast chronological aging by mechanisms that coordinate the spatiotemporal dynamics of various cellular processes before entry into a non-proliferative state and after such entry. Caloric restriction causes a stepwise establishment of an aging-delaying cellular pattern by tuning a network that assimilates the following: 1) pathways of carbohydrate and lipid metabolism; 2) communications between the endoplasmic reticulum, lipid droplets, peroxisomes, mitochondria and the cytosol; and 3) a balance between the processes of mitochondrial fusion and fission. Through different phases of the aging process, the caloric restriction-dependent remodeling of this intricate network 1) postpones the age-related onsets of apoptotic and liponecrotic modes of regulated cell death; and 2) actively increases the chance of cell survival by supporting the maintenance of cellular proteostasis. Because caloric restriction decreases the risk of cell death and actively increases the chance of cell survival throughout chronological lifespan, this dietary intervention extends longevity of chronologically aging yeast.

## INTRODUCTION

Caloric restriction (CR), a diet in which only the intake of calories is lowered whereas the supply of amino acids, vitamins and other nutrients is not limited, delays aging and postpones the onset of age-related diseases in evolutionarily distant multicellular eukaryotes [1–3]. CR also slows down the replicative and chronological modes of aging in the budding yeast *Saccharomyces cerevisiae* [2, 4–7], a unicellular eukaryote successfully used as a model organism for uncovering mechanisms of aging and aging delay [2, 4–10]. Our studies of how CR influences a pattern of metabolism and organelle dynamics in chronologically aging yeast have revealed that this low-calorie diet alters age-related dynamics of certain cellular processes [4, 6, 7, 9, 11, 12]. Among these cellular processes are ethanol metabolism, lipid synthesis and degradation, trehalose metabolism, reactive oxygen species (ROS) homeostasis maintenance, mitochondrial morphology control, mitochondrial functionality preservation, stress response control, cell cycle regulation, quiescence maintenance, and apoptotic and liponecrotic death subroutines [4, 6, 7, 9, 11, 12]. It remains unclear how CR coordinates the spatiotemporal dynamics of all these cellular processes to delay yeast chronological aging.

In these study, we investigated mechanisms through which CR orchestrates various cellular processes to slow down yeast chronological aging. We show that CR extends longevity of chronologically aging yeast by remodeling carbohydrate and lipid metabolism, affecting certain interorganellar communications, changing morphologies and functional states of peroxisomes and mitochondria, and delaying the age-related onsets of apoptotic and liponecrotic forms of regulated cell death (RCD).

## RESULTS

### Rapid consumption of ethanol by yeast cultured under CR conditions is an essential contributing factor to chronological aging delay by CR

We have previously found that wild-type (WT) cells of yeast grown under CR conditions on 0.2% or 0.5% glucose quickly consume ethanol, a product of glucose fermentation by these cells [4]. Because WT yeast cultures grown under non-CR conditions on 1% or 2% glucose did not consume (and therefore accumulated) ethanol for many days of culturing [4], we hypothesized that the fast consumption of ethanol by WT yeast limited in calorie supply may play essential role in the ability of CR to delay yeast chronological aging [4, 6, 11]. In our hypothesis, ethanol accumulation by yeast cells cultured in calorie-rich medium may be responsible for the accelerated chronological aging of non-CR yeast [4, 6, 11]. This hypothesis posits that: 1) a genetic intervention that lowers ethanol concentration in non-CR yeast cultures will extend longevity of chronologically aging yeast; whereas 2) a genetic intervention that rises ethanol concentration in such cultures will shorten yeast chronological lifespan (CLS) [4, 6, 11]. To test this hypothesis, we assessed how a single-gene-deletion mutation eliminating either the Adh1 or the Adh2 isozyme of alcohol dehydrogenase influences ethanol concentrations in CR and non-CR yeast cultures and also how each of these mutations affects CLS of CR and non-CR yeast. Adh1 is known to catalyze acetaldehyde conversion to ethanol, whereas Adh2 is involved in the reverse process of ethanol conversion to acetaldehyde (Supplementary Figure 1) [13]. Both these alcohol dehydrogenase isozymes are assimilated into a network of metabolic pathways and interorganellar communications taking place in cells of chronologically aging *S. cerevisiae* (Supplementary Figure 1) [4, 6, 9, 13–16].

**Figure 1:**
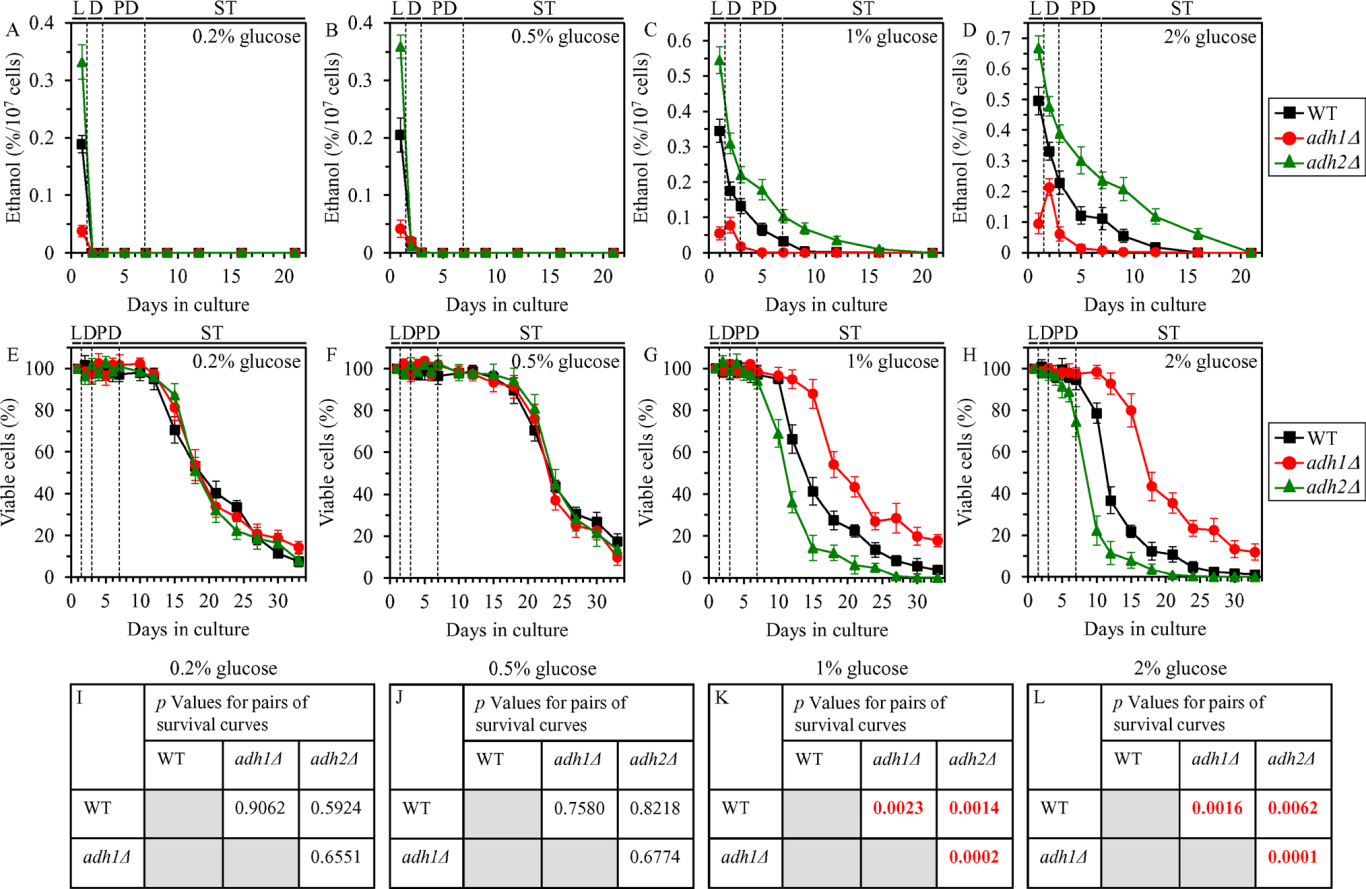
Effects of the *adh1Δ* and *adh2Δ* mutations on ethanol concentration and CLS under CR and non-CR conditions WT, *adh1Δ* and *adh2Δ* cells were cultured in the nutrient-rich YP medium under CR (the initial concentration of glucose was 0.2% or 0.5%) or non-CR (the initial concentration of glucose was 1% or 2%) conditions. (**A**–**D**) Ethanol concentrations in yeast cultures grown under CR or non-CR conditions and recovered on different days of culturing. (**E**–**H**) Survival curves of chronologically aging WT, *adh1Δ* and *adh2Δ* strains. Data are presented as means ± SEM (*n* = 4). (**I**–**L**) *p* Values for different pairs of survival curves of WT, *adh1Δ* and *adh2Δ* strains cultured under CR or non-CR conditions. Survival curves shown in (E–H) were compared. Two survival curves were considered statistically different if the *p* value was less than 0.05. The *p* values for comparing pairs of survival curves using the logrank test were calculated as described in “Materials and Methods”. Abbreviations: L, D, PD and ST, logarithmic, diauxic, post-diauxic and stationary growth phases (respectively).

We found that, although the *adh1Δ* mutation decreases and the *adh2Δ* mutation increases ethanol concentration in yeast cultures during logarithmic (L) phase of growth under CR on 0.2% or 0.5% glucose, ethanol is rapidly and completely consumed by *adh1Δ* and *adh2Δ* cells (as well as by WT cells) during subsequent diauxic (D) phase of culturing under CR conditions (Figure 1A and 1B, respectively). Not only the *adh1Δ* and *adh2Δ* mutations had no effect on the rapid consumption of ethanol under CR conditions, but they also did not affect the CLS of yeast placed on a low-calorie diet (Figure 1E, 1F, 1I and 1J).

Despite the *adh1Δ* and *adh2Δ* mutations did not influence ethanol concentration in post-logarithmic CR yeast cultures and had no effect on yeast CLS under CR conditions, each of them differently affected ethanol concentration and CLS under non-CR conditions on 1% or 2% glucose. *adh1Δ* significantly decreased ethanol concentration in non-CR yeast during all phases of culturing (Figure 1C and 1D) and also increased the CLS of non-CR yeast (Figure 1G, 1H, 1K and 1L). In contrast, *adh2Δ* significantly increased ethanol concentration in non-CR yeast during all phases of culturing (Figure 1C and 1D) and also decreased the CLS of non-CR yeast (Figure 1G, 1H, 1K and 1L).

In sum, these findings indicate that CR delays yeast chronological aging in part because this low-calorie diet accelerates the consumption of ethanol, a product of glucose fermentation.

### Ethanol concentration controls the homeostasis of glycogen and trehalose in yeast cultured under non-CR conditions

Both the Adh1-dependent conversion of acetaldehyde to ethanol and the Adh2-driven conversion of ethanol to acetaldehyde are integrated into a network which includes the metabolic pathways for glycogen and trehalose synthesis and degradation (Supplementary Figure 1) [4, 6, 13, 17]. Glycogen and trehalose are the major stores of glucose in yeast cells [4, 6, 17, 18]. Trehalose in yeast is also involved in the development and maintenance of an anti-aging cellular pattern because this carbohydrate: 1) protects cells and proteins from various stresses [19]; 2) preserves cellular proteostasis by suppressing the misfolding, aggregation and oxidative damage of newly synthesized polypeptides [6, 12, 20]; and 3) enables a re-entry of quiescent cells into the proliferative cell cycle in response to a supply of nutrients [7, 21, 22].

Because the pathways of ethanol, glycogen and trehalose metabolism are integrated into a network and play essential roles in yeast chronological aging, we hypothesized that the extent of ethanol accumulation by yeast cells cultured under non-CR conditions may influence glycogen and/or trehalose homeostasis in yeast cells. Our hypothesis posits that genetic interventions that decrease or increase ethanol concentration in non-CR yeast cultures will differently affect the concentrations of glycogen and/or trehalose in yeast cells cultured in calorie-rich medium. To test this hypothesis, we examined how the *adh1Δ* mutation (which decreases ethanol concentration in non-CR yeast; Figure 1C and 1D) and the *adh2Δ* mutation (which increases ethanol concentration in non-CR yeast; Figure 1C and 1D) affect glycogen and trehalose abundance in yeast cultured under non-CR conditions.

**Figure 2:**
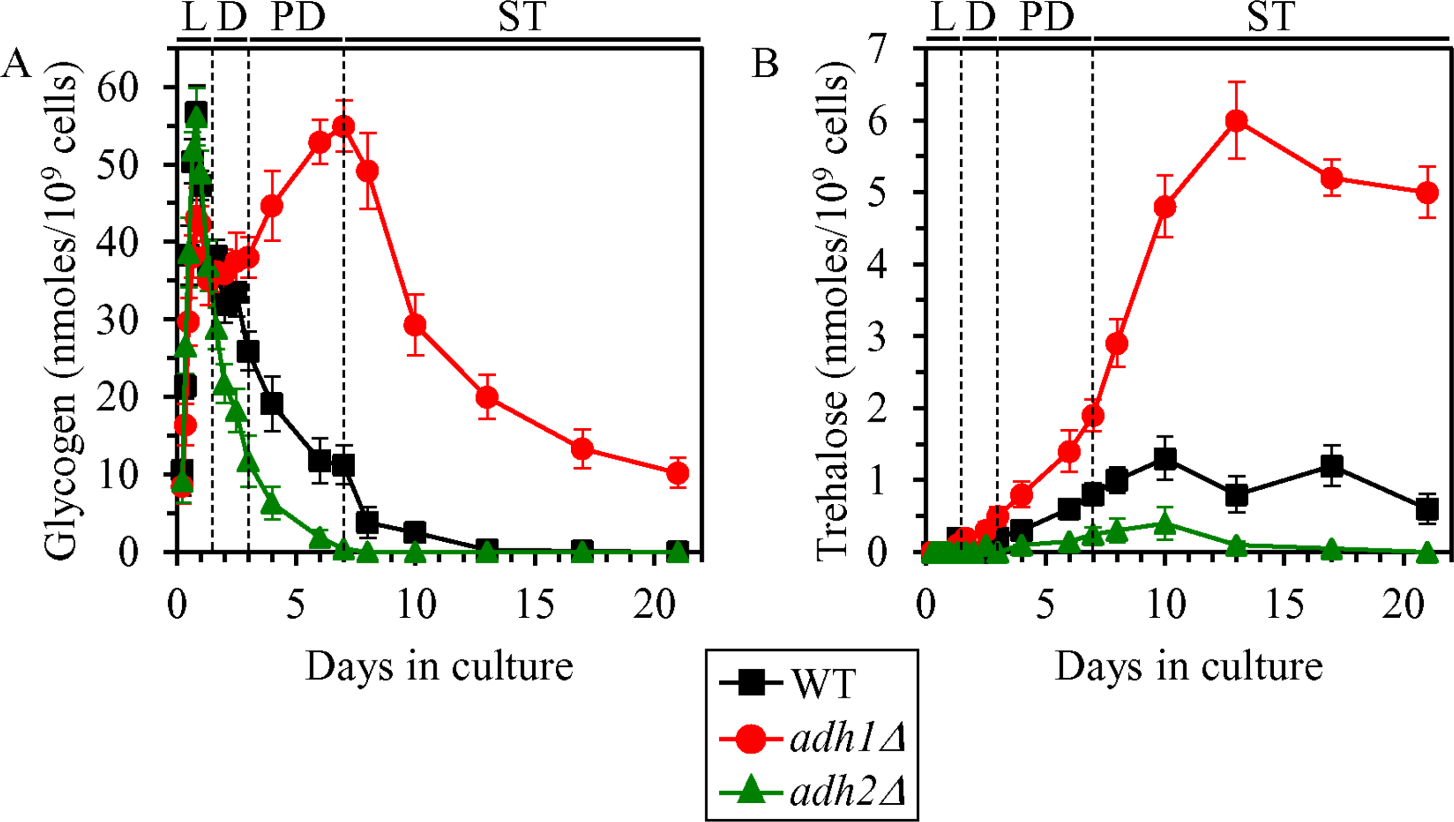
Effects of the *adh1Δ* and *adh2Δ* mutations on glycogen and trehalose concentrations in non-CR yeast WT, *adh1Δ* and *adh2Δ* cells were cultured in the nutrient-rich YP medium under non-CR conditions on 2% glucose. Glycogen (**A**) and trehalose (**B**) concentrations in non-CR yeast recovered on different days of culturing are shown. Glycogen and trehalose concentrations were measured as described in “Materials and Methods”. Abbreviations: L, D, PD and ST, logarithmic, diauxic, post-diauxic and stationary growth phases (respectively).

We found that the *adh1Δ* mutation has the following effects on glycogen and trehalose in non-CR yeast cultured on 2% glucose: 1) it elicits a buildup of glycogen during post-diauxic (PD) phase of culturing and shifts glycogen consumption by *adh1Δ* cells to stationary (ST) phase (whereas WT cells deplete glycogen store during preceding PD phase) (Figure 2A); and 2) it substantially increases the concentration of trehalose during PD and ST phases (Figure 2B). In contrast, the *adh2Δ* mutation affected glycogen and trehalose abundance in non-CR yeast cultured on 2% glucose as follows: 1) it accelerated the consumption of glycogen during D and PD phases (Figure 2A); and 2) it significantly decreased trehalose concentration during PD and ST phases (Figure 2B).

These findings support our hypothesis that ethanol concentration in non-CR yeast cells regulates glycogen and trehalose homeostasis in these cells.

### Ethanol concentration regulates the homeostasis of neutral lipids, free fatty acids and diacylglycerols, in non-CR yeast

The network integrating ethanol, glycogen and trehalose metabolism also incorporates the metabolic pathways for the synthesis and degradation of the following lipid classes: 1) the so-called ″neutral″ (uncharged) lipids triacylglycerols (TAG) and ergosteryl esters (EE), both of which are first synthesized in the endoplasmic reticulum (ER) and then deposited in lipid droplets (LD); and 2) free fatty acids (FFA) and diacylglycerols (DAG), both of which can be used for TAG synthesis in the ER and can also be formed as the products of TAG lipolysis in LD (FFA can also undergo β-oxidation in peroxisomes) (Supplementary Figure 1) [4, 6, 14–16]. TAG synthesis from FFA and DAG in the ER, LD-confined TAG lipolysis into FFA and DAG, and the peroxisomal oxidation of FFA are known to be longevity assurance processes in chronologically aging yeast [4, 6, 9, 14–16].

Given that the metabolic pathways for ethanol, TAG, EE, FFA and DAG synthesis and degradation are assimilated into a network and define longevity of chronologically aging yeast, one may assume that ethanol concentration in non-CR yeast may play an important role in the maintenance of TAG, EE, FFA and DAG homeostasis. To test this assumption, we assessed the effects of the *adh1Δ* and *adh2Δ* mutations on the abundance of LD, TAG, EE, FFA and DAG in non-CR yeast.

**Figure 3:**
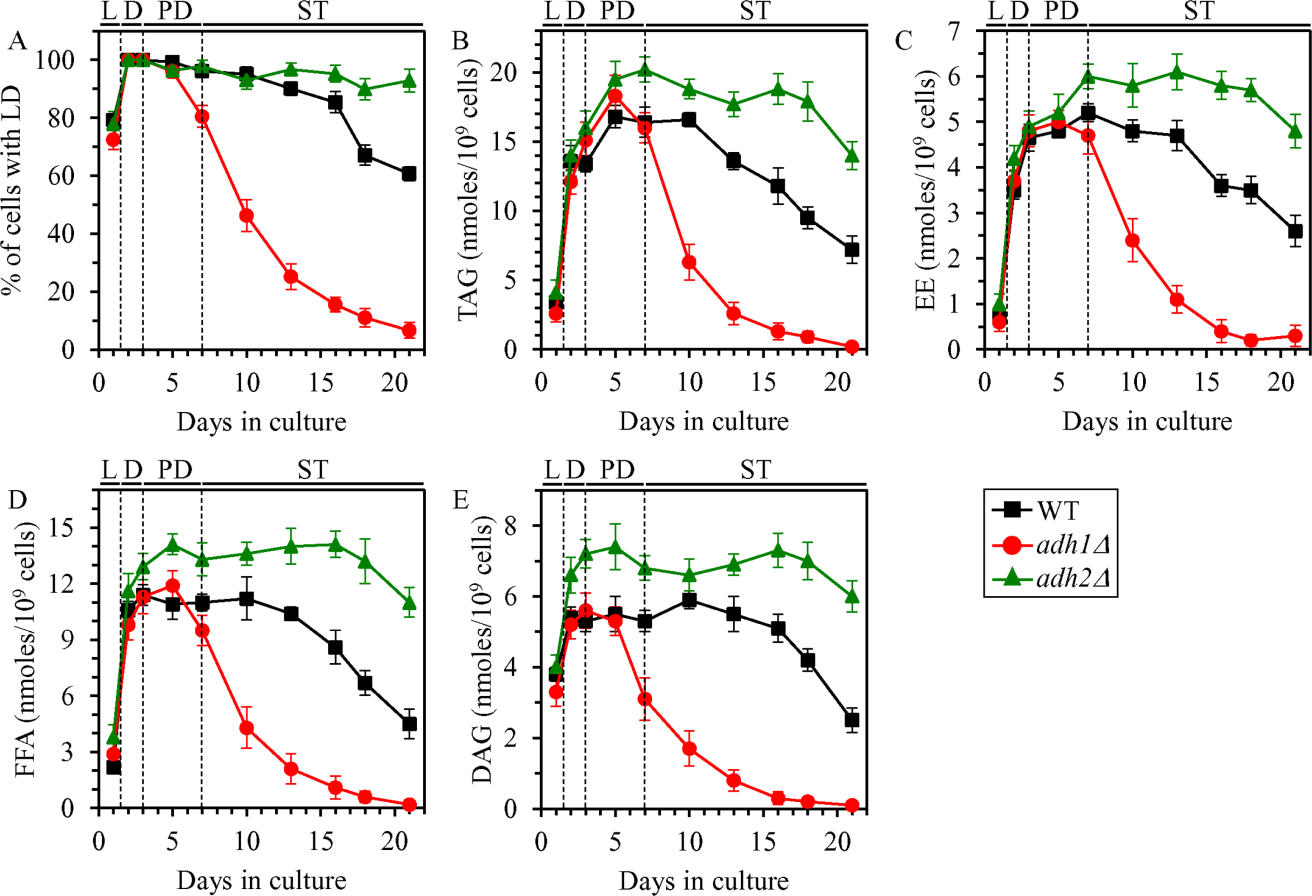
Effects of the *adh1Δ* and *adh2Δ* mutations on the abundance of LD, TAG, EE, FFA and DAG in non-CR yeast WT, *adh1Δ* and *adh2Δ* cells were cultured in the nutrient-rich YP medium under non-CR conditions on 2% glucose. The percentage of cells with LD (**A**) as well as TAG (**B**), EE (**C**), FFA (**D**) and DAG (**E**) concentrations in whole cells recovered on different days of culturing are shown. The abundance of LD and the concentrations of TAG, EE, FFA and DAG in whole cells were measured as described in “Materials and Methods”. Abbreviations: DAG, diacylglycerols; EE, ergosteryl esters; FFA, free fatty acids; LD, lipid droplets; L, D, PD and ST, logarithmic, diauxic, post-diauxic and stationary growth phases (respectively); TAG, triacylglycerols.

We found that in yeast cultured under non-CR conditions on 2% glucose, the *adh1Δ* mutation stimulates LD degradation, promotes TAG and EE lipolysis, and also accelerates FFA and DAG consumption during ST phase (Figure 3A–3E); this mutation decreases ethanol concentration in non-CR yeast (Figure 1C and 1D). We also noticed that the *adh2Δ* mutation has the following effects in non-CR yeast cultured on 2% glucose: 1) it suppresses LD degradation during late ST phase; 2) it slows down TAG and EE lipolysis during ST phase; and 3) it rises FFA and DAG concentrations during PD phase and decelerates their consumption during subsequent ST phase (Figure 3A–3E); this mutation increases ethanol concentration in non-CR yeast (Figure 1C and 1D).

In sum, these findings confirm our assumption that ethanol concentration in non-CR yeast cells is an important contributing factor to the maintenance of TAG, EE, FFA and DAG homeostasis.

### Ethanol lowers concentrations of enzymes involved in the peroxisomal oxidation of FFA to cause FFA accumulation in non-CR yeast

Our previous study has revealed that in chronologically aging yeast the CR diet: 1) elicits a rapid and complete exhaustion of ethanol during D phase; 2) increases the concentrations of the core enzymes of peroxisomal fatty acid β-oxidation Fox1, Fox2 and Fox3 (Supplementary Figure 1) during D, PD and ST phases; and 3) stimulates a fast depletion of FFA during PD phase [4]. Ethanol has been shown to decrease the levels of Fox1, Fox2 and Fox3 in methylotrophic yeast capable of using methanol as sole source of carbon and energy [23, 24]. Altogether, these findings suggested a hypothesis that the observed buildup of ethanol in non-CR yeast cultures (Figure 1C and 1D) may decrease the abundance of Fox1, Fox2 and Fox3; the resulting decline in the peroxisomal β-oxidation of FFA may instigate the accumulation of FFA seen in non-CR yeast [4].

**Figure 4:**
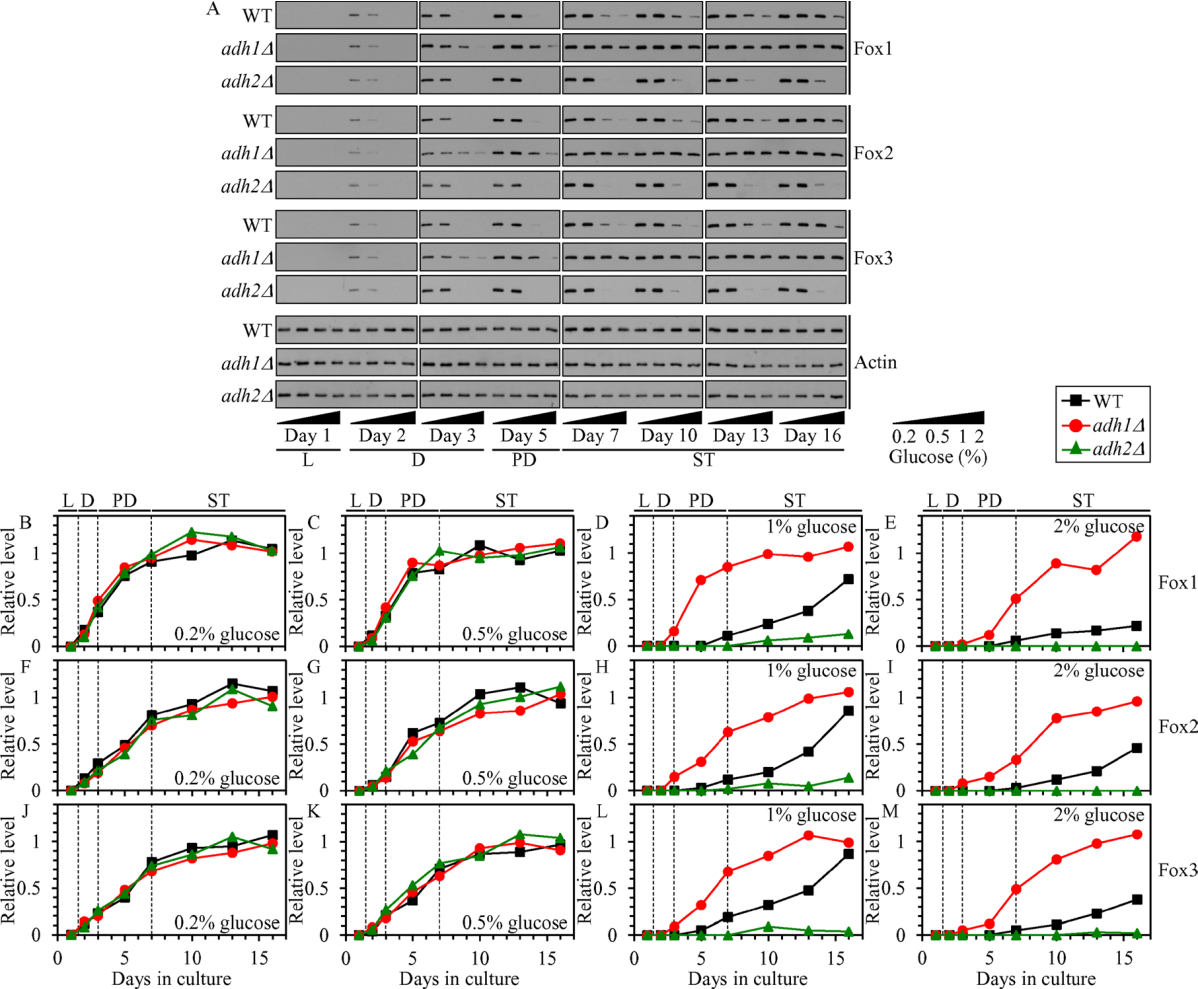
Effects of the *adh1Δ* and *adh2Δ* mutations on the concentrations of Fox1, Fox2 and Fox3 in yeast cultured under CR or non-CR conditions WT, *adh1Δ* and *adh2Δ* cells were cultured in the nutrient-rich YP medium under CR (the initial concentration of glucose was 0.2% or 0.5%) or non-CR (the initial concentration of glucose was 1% or 2%) conditions. (**A**) Western blot analysis of Fox1, Fox2, Fox3 and actin in total cell lysates was performed as described in “Materials and Methods”. (**B**–**M**) Immunoblots shown in (A) were used to calculate the relative levels of Fox1, Fox2 and Fox3 in total cell lysates as described in “Materials and Methods”. Abbreviations: L, D, PD and ST, logarithmic, diauxic, post-diauxic and stationary growth phases (respectively).

If this hypothesis is correct, then: 1) a genetic intervention that decreases ethanol concentration in non-CR yeast cultures (such as the *adh1Δ* mutation) will increase the levels of Fox1, Fox2 and Fox3 in yeast cultured in calorie-rich medium; whereas 2) a genetic intervention that increases ethanol concentration in these cultures (such as the *adh2Δ* mutation) will decrease the levels of Fox1, Fox2 and Fox3 in non-CR yeast. In support of this hypothesis, the *adh1Δ* mutation caused an increase in the concentrations of Fox1, Fox2 and Fox3 in non-CR yeast cultured on 1% or 2% glucose, while the *adh2Δ* mutation had the opposite effect on the concentrations of these enzymes in yeast under non-CR conditions (Figure 4A–4M).

We therefore concluded that ethanol decreases the concentrations of Fox1, Fox2 and Fox3 in non-CR yeast cells, thus decelerating the peroxisomal β-oxidation of FFA and eliciting a buildup of FFA in these cells.

### In CR yeast, the peroxisomal β-oxidation of FFA is a longevity assurance process that controls neutral lipids synthesis in the ER and neutral lipids lipolysis in LD

**Figure 5:**
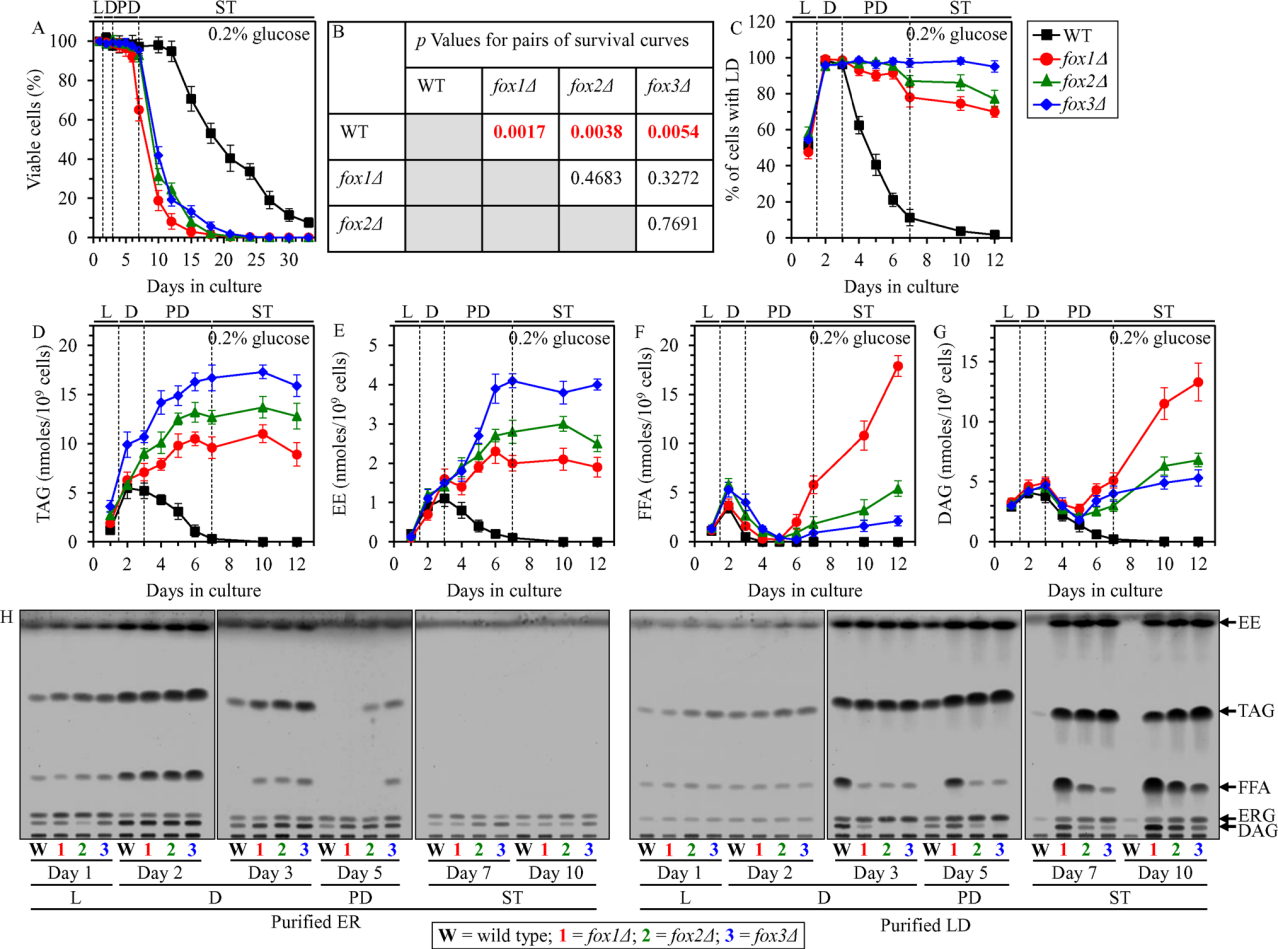
Effects of the *fox1Δ*, *fox2Δ* and *fox3Δ* mutations on CLS, LD abundance, and TAG, EE, FFA and DAG concentrations in yeast cultured under CR conditions WT, *fox1Δ*, *fox2Δ* and *fox3Δ* cells were cultured in the nutrient-rich YP medium under CR conditions on 0.2% glucose. (**A**) Survival curves of chronologically aging WT, *fox1Δ*, *fox2Δ* and *fox3Δ* strains. Data are presented as means ± SEM (*n* = 4). (**B**) *p* Values for different pairs of survival curves of WT, *fox1Δ*, *fox2Δ* and *fox3Δ* strains. Survival curves shown in (A) were compared. Two survival curves were considered statistically different if the *p* value was less than 0.05. The *p* values for comparing pairs of survival curves using the logrank test were calculated as described in “Materials and Methods”. The percentage of cells with LD (**C**) as well as TAG (**D**), EE (**E**), FFA (**F**) and DAG (**G**) concentrations in whole cells recovered on different days of culturing are shown. Data are presented as means ± SEM (*n* = 4). The abundance of LD and the concentrations of TAG, EE, FFA and DAG in whole cells were measured as described in “Materials and Methods”. (**H**) Spectra of lipids that were extracted from the ER or LD purified from WT, *fox1Δ*, *fox2Δ* and *fox3Δ* cells. Cells were recovered on different days of culturing, and the ER and LD were purified as described in “Materials and Methods”. The equivalent of 200 μg of ER proteins and the equivalent of 20 μg of LD proteins were used for lipid extraction and analysis by thin-layer chromatography, as described in “Materials and Methods”. A representative of 3 independent experiments is shown. Abbreviations: DAG, diacylglycerols; EE, ergosteryl esters; ER, endoplasmic reticulum; FFA, free fatty acids; LD, lipid droplets; L, D, PD and ST, logarithmic, diauxic, post-diauxic and stationary growth phases (respectively); TAG, triacylglycerols.

Based on the fact that CR diet elicits a substantial increase in the concentrations of the core enzymes of peroxisomal fatty acid β-oxidation Fox1, Fox2 and Fox3 (Figure 4) [4], one may assume that the β-oxidation of FFA in peroxisomes may be a longevity assurance process in chronologically aging yeast limited in calorie supply. In support of this assumption, we found that the *fox1Δ*, *fox2Δ* and *fox3Δ* mutations significantly shorten the CLS of yeast cultured under CR on 0.2% glucose (Figure 5A and 5B).

**Figure 6:**
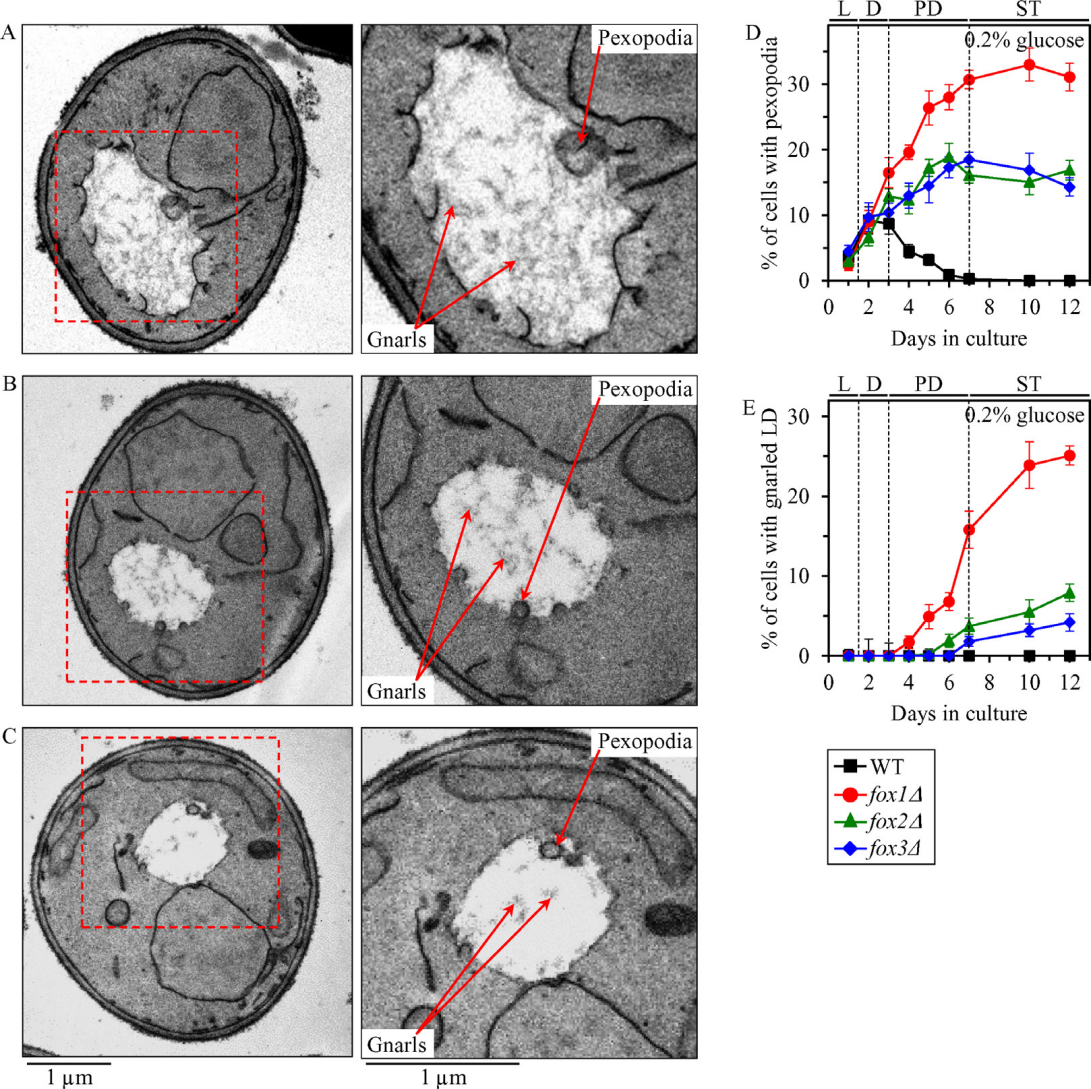
Effects of the *fox1Δ*, *fox2Δ* and *fox3Δ* mutations on age-related changes in the abundance of pexopodia and LD-confined gnarls in yeast cultured under CR conditions WT, *fox1Δ*, *fox2Δ* and *fox3Δ* cells were cultured in the nutrient-rich YP medium under CR conditions on 0.2% glucose. (**A**–**C**) Transmission electron micrographs of chronologically aging *fox1Δ* (A), *fox2Δ* (B) and *fox3Δ* (C) cells recovered on day 7. Each of the two panels for *fox1Δ*, *fox2Δ* and *fox3Δ* cells shows a different magnification of the same electron micrograph. The percentage of WT, *fox1Δ*, *fox2Δ* and *fox3Δ* cells with pexopodia (**D**) and LD-confined gnarls (**E**) in yeast recovered on different days of culturing are presented. Images similar to the representative images shown in (A, B and C) were quantified as described in “Materials and Methods”. Data are presented as means ± SEM (*n* = 3). Abbreviations: L, D, PD and ST, logarithmic, diauxic, post-diauxic and stationary growth phases (respectively).

FFA that undergo the β-oxidation in peroxisomes are formed in LD as the products of neutral lipid lipolysis, a process which also generates DAG and ergosterol (ERG) from TAG and EE, respectively, (Supplementary Figure 1) [4, 6, 14–16]. Moreover, LD-derived FFA are utilized for neutral lipids synthesis in the ER using DAG and ERG as the co-substrates for TAG and EE formation (respectively) (Supplementary Figure 1) [4, 6, 14–16]. Thus, the β-oxidation of FFA in peroxisomes, lipolysis of neutral lipids in LD and synthesis of neutral lipids in the ER are integrated into an intricate metabolic network (Supplementary Figure 1). We therefore hypothesized that the *fox1Δ*, *fox2Δ* and *fox3Δ* mutations may alter the concentrations of all these lipid classes (i.e. FFA, DAG, TAG and EE). Our hypothesis posits that the rate of the peroxisomal β-oxidation of FFA may influence the rates of neutral lipids synthesis in the ER and neutral lipids lipolysis in LD. In support of this hypothesis, we found that in chronologically aging CR yeast the *fox1Δ*, *fox2Δ* and *fox3Δ* mutations 1) prevent an age-related depletion of LD during PD and ST phases (Figure 5C); 2) eliminate an age-related decline in the cellular concentrations of TAG and EE during PD and ST phases (Figure 5D and 5E); 3) elicit an accumulation of TAG and EE in the ER during PD phase (Figure 5H); 4) cause a buildup of TAG and EE in LD during ST phase (Figure 5H); 5) increase the cellular concentrations of FFA and DAG during PD and ST phases (Figure 5F and 5G); 6) prompt an accumulation of FFA and DAG in the ER during D and PD phases (Figure 5H); and 7) promote a deposition of FFA and DAG in LD during PD and ST phases (Figure 5H).

### A weakening of peroxisomal fatty acid β-oxidation elicits negative feedback loops that regulate the metabolism and transport of several lipid classes in the ER and LD

**Figure 7:**
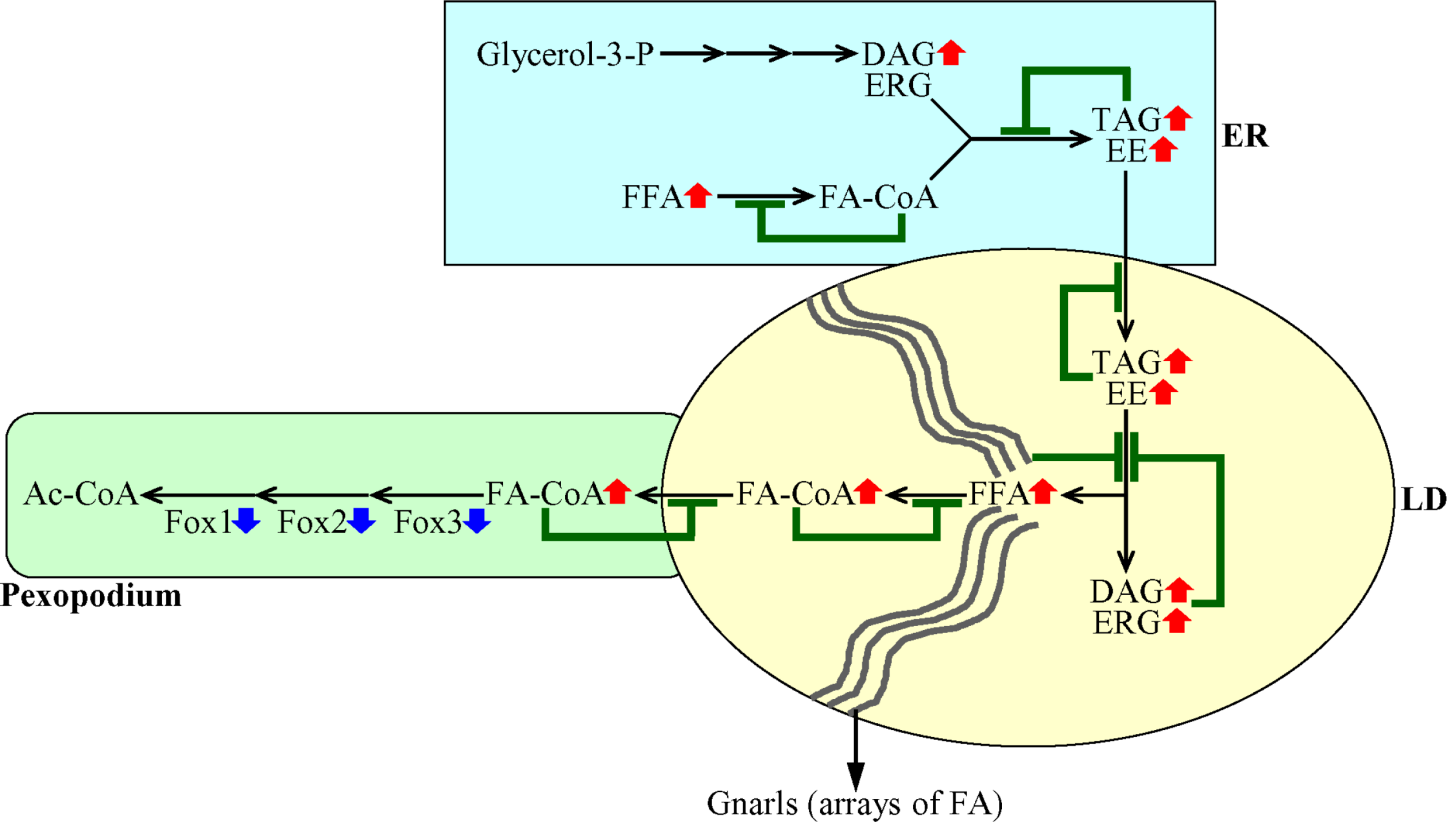
A mechanism through which a decline in the peroxisomal β-oxidation of FFA elicits negative feedback loops that regulate the metabolism and transport of several lipid classes in the ER and LD A weakening of the peroxisomal β-oxidation of FFA causes an accumulation of FA-CoA in pexopodia, which represent intrusions of peroxisomes into the neutral lipid core of LD. This creates several negative feedback loops whose action ultimately causes an age-related build-up of FFA and DAG in the ER and LD. Blue arrows next to the names of Fox1, Fox2 and Fox3 indicate that a decline in the peroxisomal β-oxidation of FFA is due to a decrease in the concentrations of the core enzymes of such oxidation. Red arrows next to the names of lipid classes denote those of them whose concentrations are increased. Inhibition bars displayed in green color signify negative feedback loops. See text for more details. Abbreviations: Ac-CoA, acetyl-CoA; DAG, diacylglycerols; EE, ergosteryl esters; ERG, ergosterol; ER, endoplasmic reticulum; FA-CoA, acyl-CoA esters; FFA, free fatty acids; LD, lipid droplets; LD, lipid droplet; TAG, triacylglycerols.

A close association of peroxisomes with LD in chronologically aging yeast causes an intrusion of peroxisomes into the neutral lipid core of LD, thus forming so-called ″pexopodia″ (Figure 6A–6C; Figure 7) [25]. Pexopodia stimulate the lipolysis of neutral lipids within LD, thereby increasing the supply of FFA for β-oxidation in peroxisomes [25]. The *fox1Δ*, *fox2Δ* and *fox3Δ* mutations impair peroxisomal fatty acid β-oxidation, thus eliciting a deposition of electron-dense arrays of FFA (termed ″gnarls″) and non-degraded neutral lipids within LD (Figure 6A–6C; Figure 7) [25].

We have used electron microscopy to monitor age-related changes in the abundance of pexopodia and LD-confined gnarls in WT, *fox1Δ*, *fox2Δ* and *fox3Δ* cells cultured under CR on 0.2% glucose.

We found that pexopodia amass in WT, *fox1Δ*, *fox2Δ* and *fox3Δ* cells during L and D phases (Figure 6D), concomitantly with an increase of FFA and DAG in whole cells (Figure 5F and 5G), the ER (Figure 5H) and LD (Figure 5H).

During the following PD phase, pexopodia disappeared in WT cells (Figure 6D); such disappearance of pexopodia in WT cells during PD phase coincided with 1) a depletion of FFA and DAG in whole WT cells (Figure 5F and 5G), the ER of WT (Figure 5H) and LD of WT (Figure 5H); 2) an exhaustion of TAG and EE in whole WT cells (Figure 5D and 5E) and the ER of WT (Figure 5H); 3) a decrease of TAG and EE concentrations in LD of WT (Figure 5H; both TAG and EE were completely depleted in LD of WT by the beginning of the ensuing ST phase); and 4) a lack of gnarls in LD of WT (Figure 6E). In contrast, pexopodia became more abundant in *fox1Δ*, *fox2Δ* and *fox3Δ* mutant cells during PD phase (Figure 6D), concomitantly with 1) a rise of FFA and DAG in whole mutant cells (Figure 5F and 5G), the ER of mutants (Figure 5H) and LD of mutants (Figure 5H); 2) an increase in TAG and EE concentrations in whole mutant cells (Figure 5D and 5E), the ER of mutants (Figure 5H) and LD of mutants (Figure 5H); and 3) an expansion of gnarls in LD of mutants (Figure 6E).

During the subsequent ST phase, the abundance of pexopodia in *fox1Δ*, *fox2Δ* and *fox3Δ* mutant cells remained unchanged (Figure 6D), whereas the abundance of gnarls within LD of these cells significantly increased (Figure 6E). Such deposition of gnarls within LD of *fox1Δ*, *fox2Δ* and *fox3Δ* mutant cells during ST phase coincided with 1) a rise of FFA and DAG in whole mutant cells (Figure 5F and 5G) and LD of mutants (Figure 5H); 2) an increase of ERG in LD of mutants (Figure 5H); 3) an absence of FFA in the ER of mutants (Figure 5H); 4) a slight increase in DAG concentrations in the ER of mutants (Figure 5H); and 5) a lack of significant changes in TAG and EE concentrations in whole mutant cells (Figure 5D and 5E), the ER of mutants (Figure 5H) and LD of mutants (Figure 5H).

In sum, these findings suggest that a decline in the peroxisomal β-oxidation of FFA causes a build-up of fatty acyl-CoA esters (FA-CoA) in pexopodia, which represent intrusions of peroxisomes into the neutral lipid core of LD. This creates a negative feedback loop which mitigates the transport of FA-CoA to pexopodia from associated LD, where these FA-CoA are formed from FFA derived from neutral lipids (Figure 7). This, in turn, elicits an accumulation of gnarls (the electron-dense arrays of FFA [25]) in LD, thereby instigating a series of negative feedback loops. These feedback loops attenuate the lipolysis of neutral lipids in LD, transport of neutral lipids from the ER to LD, and synthesis of neutral lipids synthesis from FFA, DAG and ERG in the ER (Figure 7). Due to the action of such negative feedback mechanism regulating lipid metabolism and transport in several organelles, a decline in peroxisomal fatty acid β-oxidation in chronologically aging yeast causes an age-related build-up of FFA and DAG in the ER and LD (Figure 7).

### Three possible mechanisms through which peroxisomal fatty acid β-oxidation may define yeast CLS

As we have found, the β-oxidation of FFA in peroxisomes is a longevity assurance process in chronologically aging yeast cultured under CR-conditions (Figure 5A and 5B). It is conceivable that there may be at least three different mechanisms by which peroxisomal fatty acid β-oxidation defines longevity of chronologically aging yeast. These possible mechanisms are outlined below.

First mechanism: the final product of peroxisomal fatty acid β-oxidation is acetyl-CoA, which is transported to mitochondria after being formed in peroxisomes (Supplementary Figure 2). Mitochondrial oxidation of the peroxisomally generated pool of acetyl-CoA through the TCA cycle provides reducing equivalents for the synthesis of ATP via oxidative phosphorylation [4, 6, 13, 17]. Acetyl-CoA that is used for the synthesis of ATP in mitochondria is also made within these organelles as the product of oxaloacetate and acetate oxidation (Supplementary Figure 2) [4, 6, 13, 17]. It is feasible that an ample amount of ATP is generated in mitochondria of CR yeast because of the oxidation of acetyl-CoA made in peroxisomes, not due to the oxidation of acetyl-CoA produced in mitochondria. Thus, peroxisomal fatty acid β-oxidation may regulate longevity-defining processes inside and outside of mitochondria and influence yeast CLS under CR conditions because such oxidation may be responsible for the synthesis of the bulk of cellular ATP (Supplementary Figure 2).

Second mechanism: mitochondrial oxidation of the peroxisomally produced pool of acetyl-CoA may also be essential for the demonstrated ability of yeast mitochondria to maintain the shape of a tubular network under CR conditions [4]; in chronologically aging non-CR yeast, this network is fragmented into individual mitochondria [4]. Because mitochondrial morphology depends on a balance between the processes of mitochondrial fission and fusion [27, 28], it is conceivable that mitochondrial oxidation of peroxisomally produced acetyl-CoA in CR yeast may allow to shift this balance toward fusion (Supplementary Figure 2). Moreover, a shift of this balance toward the opposing process of mitochondrial fission elicits mitochondrial fragmentation and promotes a mitochondria-controlled apoptotic mode of age-related RCD (Supplementary Figure 2) [29–38]. Thus, it is also plausible that mitochondrial oxidation of peroxisomally produced acetyl-CoA may be essential for the demonstrated ability of CR [4, 7] to delay the onset of the apoptotic mode of age-related RCD.

Third mechanism: we have revealed that 1) a decline in peroxisomal fatty acid β-oxidation in chronologically aging yeast causes an age-related build-up of FFA and DAG in the ER and LD (Figure 7); and 2) CR stimulates peroxisomal fatty acid β-oxidation and decreases the concentrations of FFA and DAG in chronologically aging yeast [4, 39]. An exposure of yeast cells to exogenous FFA and DAG is known to elicit a ″liponecrotic″ mode of age-related RCD [39–42]. Therefore, it is possible that the abilities of CR to stimulate peroxisomal fatty acid β-oxidation and to decrease the concentrations of FFA and DAG may delay the age-related onset of the liponecrotic mode of RCD (Supplementary Figure 2).

In a series of experiments outlined below, we assessed how each of the three mechanisms contributes to the extension of yeast CLS by CR.

### The β-oxidation of FFA in peroxisomes defines the CLS of CR yeast in part because it is essential for mitochondrial functionality and ATP synthesis in mitochondria

To assess the first proposed mechanism (Supplementary Figure 2), we tested if the *fox1Δ*, *fox2Δ* and *fox3Δ* mutations can alter mitochondrial functionality and decrease the concentration of cellular ATP, which in CR yeast is produced mainly in mitochondria [15, 44]. The *fox1Δ*, *fox2Δ* and *fox3Δ* mutations deplete the peroxisomally generated pool of acetyl-CoA by impairing peroxisomal fatty acid β-oxidation (Supplementary Figure 2) [15, 44]. As we demonstrated, each of these mutations significantly shortens the CLS of yeast cultured under CR on 0.2% glucose (Figure 5A and 5B).

**Figure 8:**
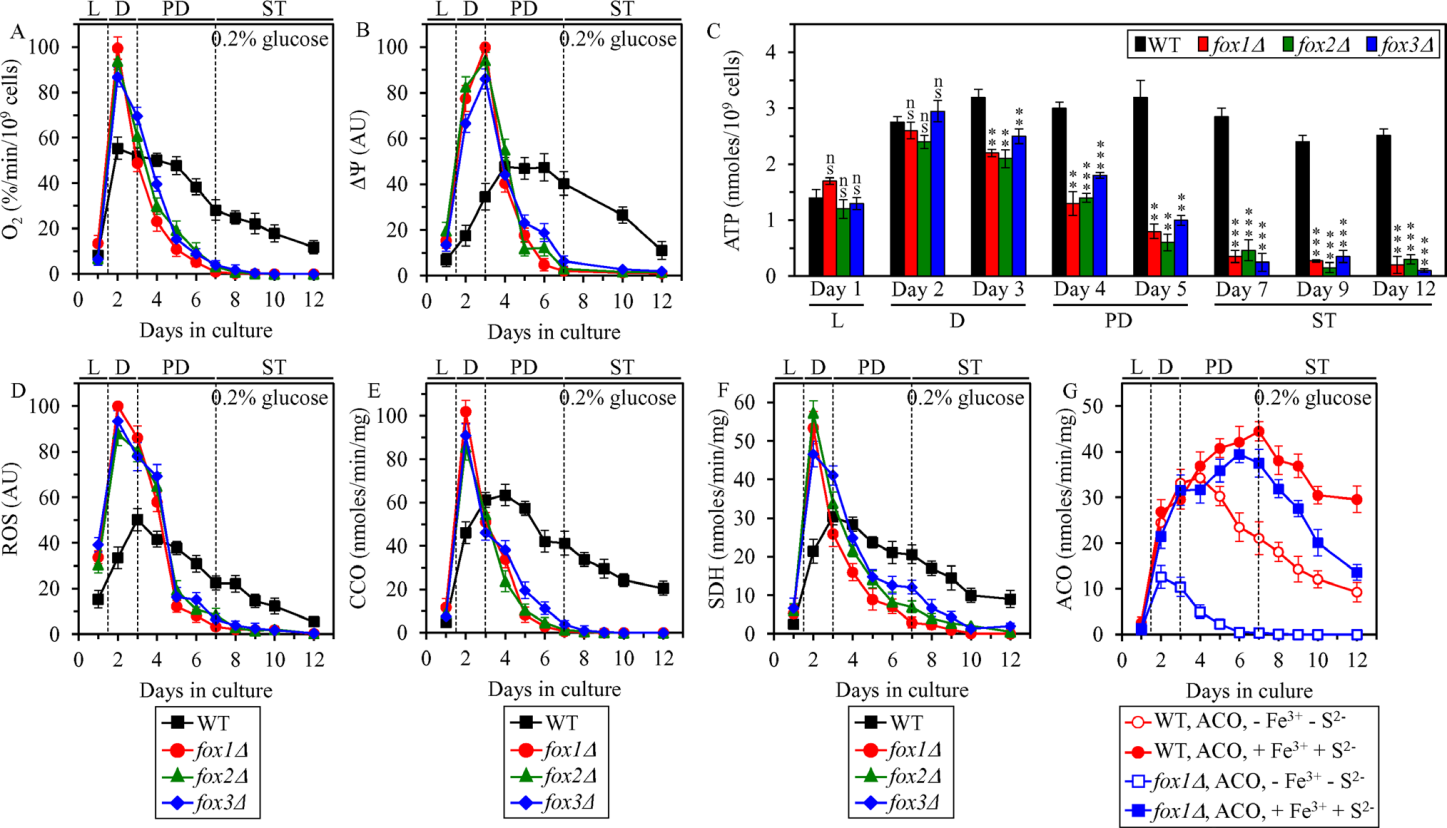
Effects of the *fox1Δ*, *fox2Δ* and *fox3Δ* mutations on vital traits of mitochondrial functionality, including ATP synthesis, in yeast cultured under CR conditions WT, *fox1Δ, fox2Δ and fox3Δ* cells were cultured in the nutrient-rich YP medium under CR conditions on 0.2% glucose. The rate of oxygen consumption by cells (**A**), electrochemical potential (*Δ*Ψ) across the IMM (**B**), cellular ATP concentration (**C**), cellular ROS concentration (**D**), enzymatic activity of cytochrome *c* oxidase (CCO) in purified mitochondria (**E**), enzymatic activity of succinate dehydrogenase in purified mitochondria (SDH) (**F**), and enzymatic activity of aconitase (ACO) in total cell lysates (**G**) were measured as described in “Materials and Methods”. The ACO activity was measured with or without the reactivation agents Fe^3+^ and Na_2_S. Data are presented as means ± SEM (*n* = 5 for (A); *n* = 3 for (B–G); ns, not significant; ^**^ < 0.01; ^***^ < 0.001). Abbreviations: L, D, PD and ST, logarithmic, diauxic, post-diauxic and stationary growth phases (respectively); IMM, inner mitochondrial membrane; ROS, reactive oxygen species.

We assessed how the *fox1Δ*, *fox2Δ* and *fox3Δ* mutations influence age-related changes in the following vital traits of mitochondrial functionality: 1) oxygen consumption by cells, which in yeast cultured in media with low (0.2%) glucose concentration is mainly due to mitochondrial respiration [13, 26]; 2) the electrochemical potential (*Δ*Ψ) across the inner mitochondrial membrane; and 3) cellular concentration of ATP, which in yeast cultured in media with low glucose concentration is generated mainly in mitochondria [13, 26]. We found that in CR yeast cultured on 0.2% glucose, the *fox1Δ*, *fox2Δ* and *fox3Δ* mutations 1) markedly amplify mitochondrial respiration (Figure 8A) and *Δ*Ψ (Figure 8B) during L and D phases; 2) elicit an abrupt decline in mitochondrial respiration (Figure 8A) and *Δ*Ψ (Figure 8B) during PD phase. Although neither of these mutations altered the concentration of mitochondrially synthesized ATP during L phase and in the beginning of D phase, all three mutations decreased ATP production in mitochondria by the end of D phase and caused a rapid decline in mitochondrial ATP synthesis during PD and ST phases (Figure 8C).

The *fox1Δ*, *fox2Δ* and *fox3Δ* mutations cause a sharp increase of mitochondrially produced ROS during L and D phases in yeast cultured under CR conditions on 0.2% glucose (Figure 8D). In agreement with an essential role that excessive concentrations of mitochondrial ROS play in oxidative damage to mitochondrial proteins [26, 43], the sharp increase of mitochondrial ROS seen in *fox1Δ*, *fox2Δ* and *fox3Δ* cells during L and D phases coincided with a rapid inactivation of several enzymes involved in the electron transport chain (ETC) and/or TCA cycle in mitochondria; such inactivation of mitochondrial ETC and TCA enzymes in *fox1Δ*, *fox2Δ* and *fox3Δ* cells continued during the subsequent PD phase (Figure 8E–8G). The protein components of mitochondrial ETC and TCA undergoing such rapid inactivation in *fox1Δ*, *fox2Δ* and *fox3Δ* cells included cytochrome *c* oxidase (CCO) (Figure 8E), succinate dehydrogenase (SDH) (Figure 8F) and aconitase (ACO) (Figure 8G), all of which are known to be highly susceptible to ROS-inflicted oxidative damage [4, 26, 43, 45, 46]. We found that the enzymatic activity of ACO, which in *fox1Δ*, *fox2Δ* and *fox3Δ* cells is rapidly decreased *in vivo* during D and PD phases, can be markedly increased *in vitro* by incubating lysates of these cells with Fe^3+^ and S^2-^ (Figure 8G); such incubation *in vitro* is known to restore the oxidative damage-dependent loss of one iron from the [4Fe-4S] cluster of ACO [47]. This observation confirms our assumption that the rapid inactivation of mitochondrial ACO seen in *fox1Δ*, *fox2Δ* and *fox3Δ* cells is due to ROS-inflicted oxidative damage to mitochondrial proteins in these cells.

Altogether, these findings support the notion that mitochondrial oxidation of acetyl-CoA pool generated in peroxisomal fatty acid β-oxidation is 1) essential for maintaining mitochondrial functionality; and 2) responsible for the synthesis of the bulk of cellular ATP fueled by the TCA cycle, ETC and oxidative phosphorylation in mitochondria.

### Peroxisomal fatty acid β-oxidation contributes to yeast CLS extension under CR conditions by weakening the fragmentation of a mitochondrial network and postponing the onset of age-related apoptotic RCD

**Figure 9:**
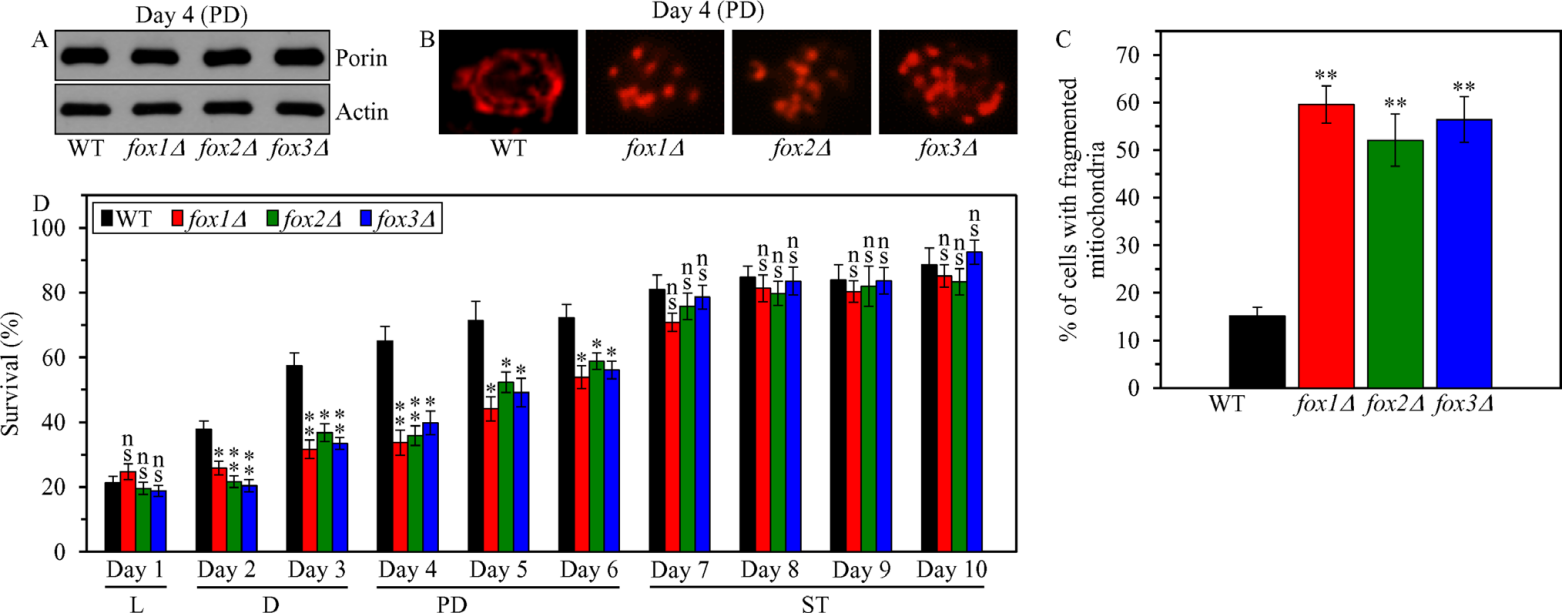
Effects of the *fox1Δ*, *fox2Δ* and *fox3Δ* mutations on mitochondrial abundance and morphology, and on cell susceptibility to mitochondria-controlled apoptotic RCD in yeast cultured under CR conditions WT, *fox1Δ, fox2Δ and fox3Δ* cells were cultured in the nutrient-rich YP medium under CR conditions on 0.2% glucose. (**A**) The concentrations of porin and actin in total cell lysates were assessed by immunoblotting, as described in “Materials and Methods”. (**B**) Mitochondrial morphology was visualized with the help of indirect immunofluorescence microscopy using primary antibodies against porin, as described in “Materials and Methods”. (**C**) The percentage of cells exhibiting fragmented mitochondria was calculated. At least 500 cells of each strain were used for quantitation. Data are presented as means ± SEM (*n* = 3; ^**^ < 0.01). (**D**) An assay for measuring clonogenic survival of cells treated for 2 h with 2.5 mM hydrogen peroxide was performed as described in “Materials and Methods”. Data are presented as means ± SEM (*n* = 3; ns, not significant; ^*^ < 0.05; ^**^ < 0.01). Abbreviations: L, D, PD and ST, logarithmic, diauxic, post-diauxic and stationary growth phases (respectively).

To assess the second proposed mechanism (Supplementary Figure 2), we initially tested if the *fox1Δ*, *fox2Δ* and *fox3Δ* mutations have an effect on the demonstrated ability [4] of yeast mitochondria to maintain the shape of a continuous tubular network under CR conditions. We found that in CR yeast cultured on 0.2% glucose 1) none of these mutations alters the concentration of porin (an abundant mitochondrial protein) and, thus, none of them influences the abundance of mitochondria (Figure 9A); and 2) each of these mutations elicits the fragmentation of the mitochondrial network into individual mitochondria during PD phase (Figure 9B and 9C), concurrently with an abrupt decline in mitochondrial functionality and ATP synthesis (Figure 8).

We then tested how the *fox1Δ*, *fox2Δ* and *fox3Δ* mutations influence the susceptibility of CR yeast to mitochondria-controlled apoptotic RCD induced by a short-term exposure of cells to exogenous hydrogen peroxide; this mode of age-related RCD has been linked to mitochondrial network fragmentation, mitochondrial outer membrane permeabilization and the efflux of several pro-apoptotic proteins from the intermembrane space of fragmented mitochondria [4, 29–38, 48]. We found that in CR yeast cultured on 0.2% glucose, each of these mutations 1) significantly decreases clonogenic survival of cells briefly (for 2 h) treated with hydrogen peroxide if these cells were recovered during D or PD phase of culturing (Figure 9D); and 2) does not alter clonogenic survival of cells subjected to such treatment if these cells were recovered during L or ST phase of culturing (Figure 9D).

These findings suggest that in CR yeast mitochondrial oxidation of acetyl-CoA that is generated in peroxisomal fatty acid β-oxidation 1) is essential for preventing mitochondrial network fragmentation during D and PD phases; and 2) is indispensable for the CR-dependent delay of the onset of age-related apoptotic RCD during these phases of culturing.

**Figure 10:**
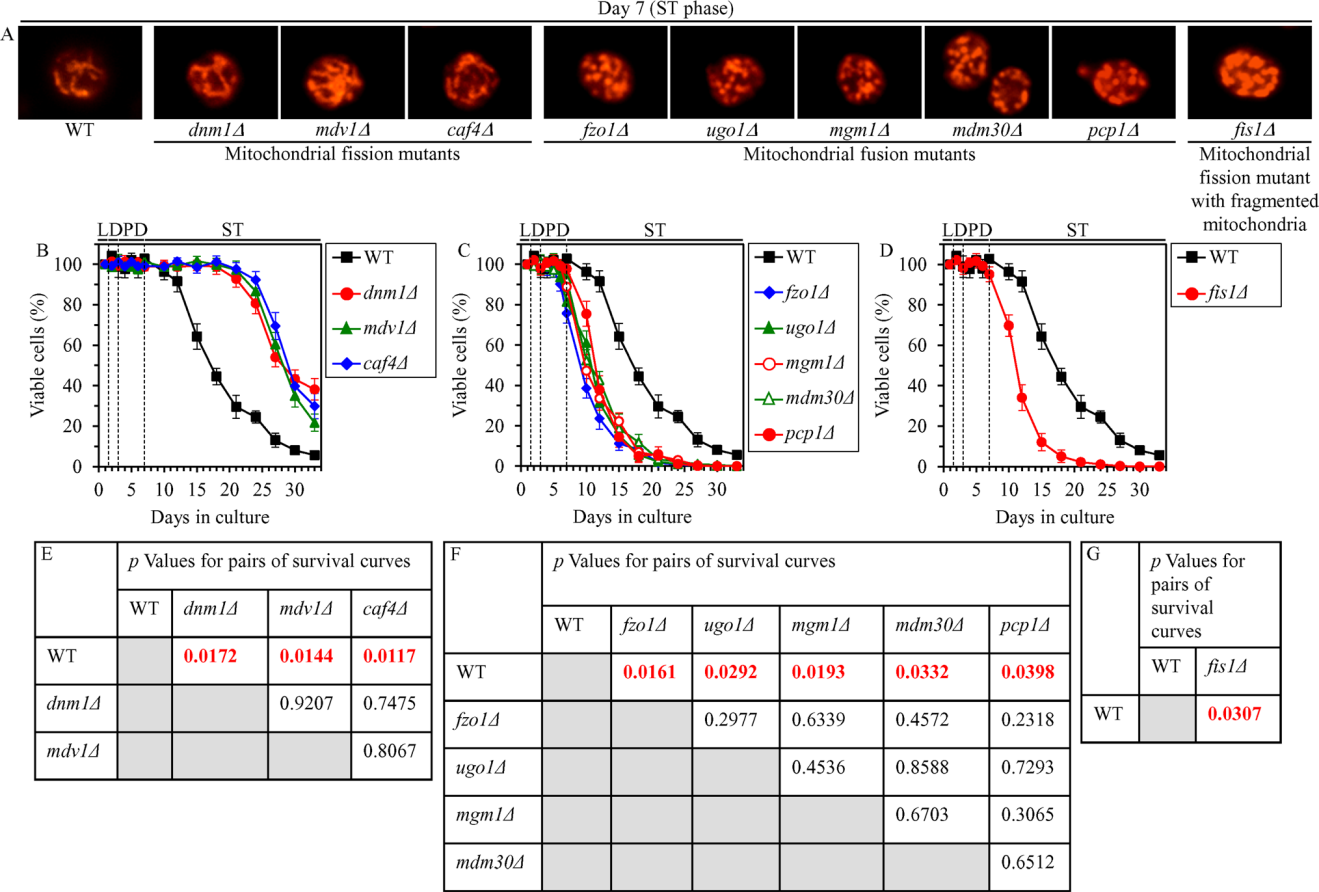
Effects of the single-gene-deletion mutations eliminating different protein components of the mitochondrial fission or fusion machine on mitochondrial morphology and CLS in yeast cultured under CR conditions WT, *dnm1Δ*, *mdv1Δ*, *caf4Δ*, *fzo1Δ*, *ugo1Δ*, *mgm1Δ*, *mdm30Δ*, *pcp1Δ* and *fis1Δ* cells were cultured in the nutrient-rich YP medium under CR conditions on 0.2% glucose. (**A**) Mitochondrial morphology was visualized with the help of indirect immunofluorescence microscopy using primary antibodies against porin, as described in “Materials and Methods”. (**B**–**D**) Survival curves of chronologically aging WT and mutant cells. Data are presented as means ± SEM (*n* = 3). (**E**–**G**) *p* Values for different pairs of survival curves of WT and mutant strains. Survival curves shown in (B–D) were compared. Two survival curves were considered statistically different if the *p* value was less than 0.05. The *p* values for comparing pairs of survival curves using the logrank test were calculated as described in “Materials and Methods”. Abbreviations: L, D, PD and ST, logarithmic, diauxic, post-diauxic and stationary growth phases (respectively).

We have previously hypothesized that the abilities of CR to attenuate mitochondrial network fragmentation and to delay the onset of a mitochondria-controlled mode of the age-related apoptotic RCD may be required for the ability of this low-calorie diet to extend yeast CLS [4, 48]. To test this hypothesis, we examined how the single-gene-deletion mutations eliminating protein components of the mitochondrial fission or fusion machine influence the extent of yeast CLS extension by CR; a balance between the processes of mitochondrial fission and fusion is known to define mitochondrial morphology [27, 28]. We found that in CR yeast cultured on 0.2% glucose 1) the *dnm1Δ*, *mdv1Δ* and *caf4Δ* mutations, which eliminate different components of the mitochondrial fission machine [33], stimulate the formation of net-like mitochondria (Figure 10A; Supplementary Figure 3) and increase the efficiency with which CR extends yeast CLS (Figure 10B and 10E); 2) the *fzo1Δ*, *ugo1Δ*, *mgm1Δ*, *mdm30Δ* and *pcp1Δ* mutations, which eliminate different components of the mitochondrial fusion machine [33], elicit mitochondrial network fragmentation (Figure 10A; Supplementary Figure 3) and decrease the efficiency of yeast CLS extension by CR (Figure 10C and 10F); and 3) the *fis1Δ* mutation, which eliminates a component of the mitochondrial fission machine (however, impairs mitochondrial fission only in exponentially growing yeast but not in yeast committed to apoptotic RCD) [33], causes mitochondrial network fragmentation (Figure 10A; Supplementary Figure 3) and lowers the efficiency with which CR extends yeast CLS (Figure 10D and 10G).

In sum, these findings support the view that mitochondrial oxidation of acetyl-CoA that is produced during peroxisomal fatty acid β-oxidation makes an essential contribution to the CR-dependent extension of yeast CLS because it averts mitochondrial network fragmentation during D and PD phases, thereby delaying the onset of an age-related mode of apoptotic RCD during these phases of culturing.

### Peroxisomal fatty acid β-oxidation contributes to the CR-dependent extension of yeast CLS in part because it slows down the onset of age-related liponecrotic RCD

**Figure 11:**
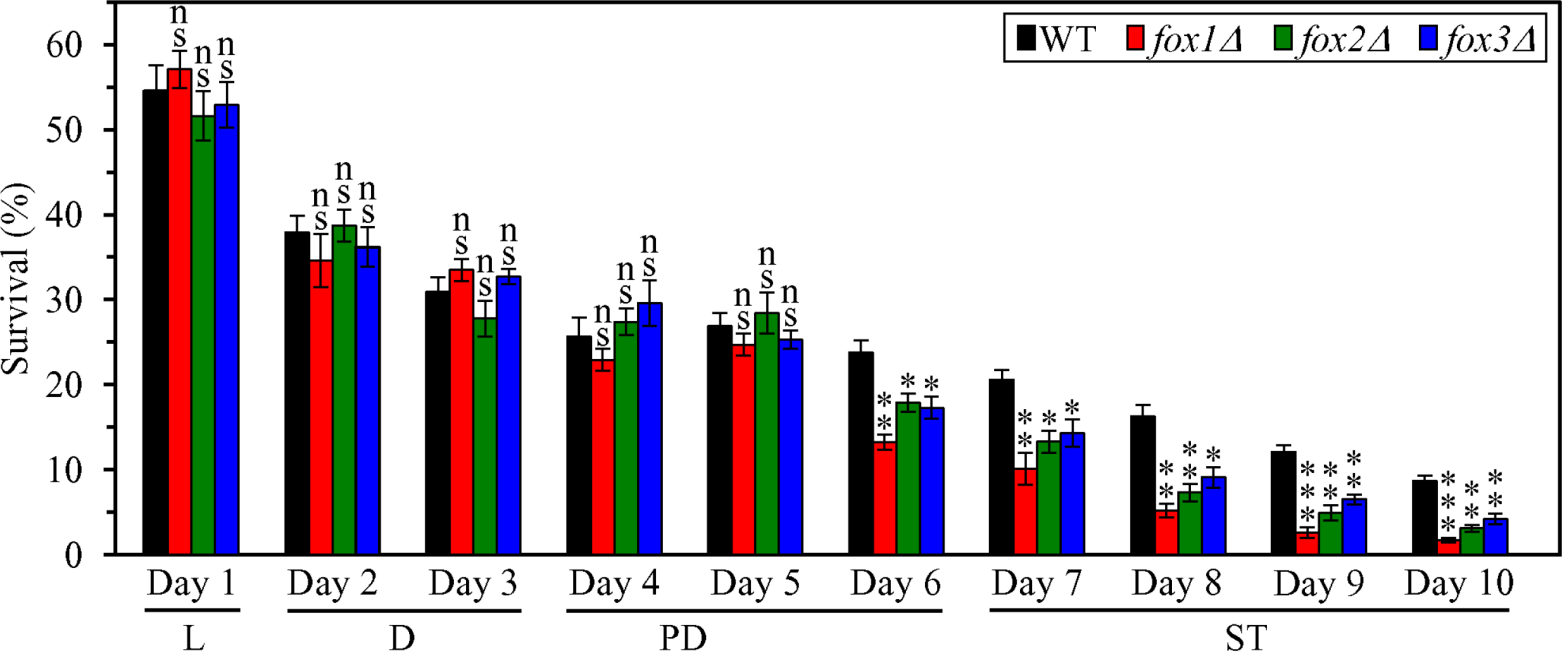
Effects of the *fox1Δ, fox2Δ* and *fox3Δ* mutations on cell susceptibility to liponecrotic RCD in yeast cultured under CR conditions WT, *fox1Δ, fox2Δ and fox3Δ* cells were cultured in the nutrient-rich YP medium under CR conditions on 0.2% glucose. An assay for measuring clonogenic survival of cells treated for 2 h with 0.15 mM palmitoleic acid, a monounsaturated FFA, was performed as described in “Materials and Methods”. Data are presented as means ± SEM (*n* = 3; ns, not significant; ^*^ < 0.05; ^**^ < 0.01; ^***^ < 0.001). Abbreviations: L, D, PD and ST, logarithmic, diauxic, post-diauxic and stationary growth phases (respectively).

To assess the third proposed mechanism (Supplementary Figure 2), we initially examined if the *fox1Δ*, *fox2Δ* and *fox3Δ* mutations affect the susceptibility of CR yeast to liponecrotic RCD; this mode of age-related RCD can be elicited by a short-term exposure of yeast cells to exogenous FFA and DAG [39–42]. We found that in CR yeast cultured on 0.2% glucose, each of these mutations 1) substantially reduces clonogenic survival of cells briefly (for 2 h) treated with palmitoleic acid (POA; a monounsaturated FFA) if these cells were recovered during late PD phase (on day 6 of culturing) or during ST phase (on days 7 to 10 of culturing) (Figure 11); and 2) has no effect on clonogenic survival of cells subjected to treatment with POA if these cells were collected during L, D or early PD phase of culturing (Figure 11). As we demonstrated, the *fox1Δ*, *fox2Δ* and *fox3Δ* mutations 2) significantly shorten the CLS of CR yeast (Figure 5A and 5B); and 2) increase the cellular concentrations of FFA and DAG during PD and ST phases (Figure 5F and 5G).

Taken together, these findings support the view that the β-oxidation of FFA in peroxisomes is necessary for the CR-dependent extension of yeast CLS in part because it allows to maintain low concentrations of FFA and DAG during PD and ST phases, thereby postponing the onset of an age-related mode of liponecrotic RCD.

We have previously hypothesized that the ability of CR to decrease the cellular concentrations of FFA and DAG during PD and ST phases may be essential for the CR-dependent extension of yeast CLS because it lowers the susceptibility of CR yeast to age-related liponecrotic RCD during these phases of culturing [4, 39–42, 48]. To test this hypothesis, we examined how the single-gene-deletion mutations eliminating redundant enzymes involved in TAG synthesis or degradation influence age-related changes in the following: 1) the concentrations of FFA, DAG and TAG in CR yeast; 2) the susceptibility of CR yeast to liponecrotic RCD elicited by a short-term treatment of cells with POA, a monounsaturated FFA; and 3) the extent of yeast CLS extension by CR. The synthesis of TAG from FFA and DAG in the ER is catalyzed by Dga1, Are1 and Are2 (Supplementary Figure 4) [14–16, 49, 50]. The lipolytic degradation of TAG into FFA and DAG in LD is catalyzed by the TAG lipases Tgl1, Tgl3, Tgl4 and Tgl5 (Supplementary Figure 4) [14–16, 51–53].

**Figure 12:**
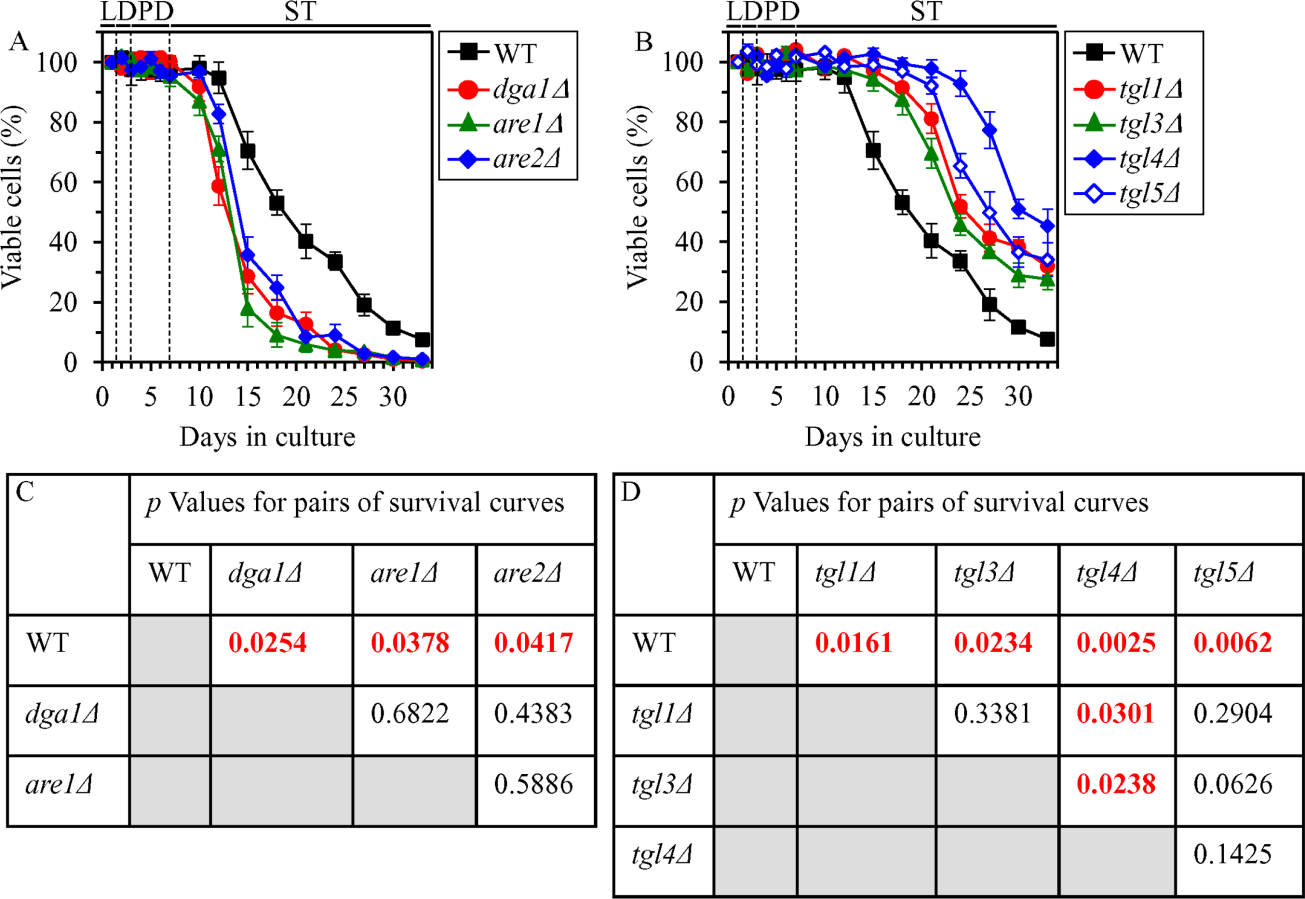
Effects of the *dga1Δ*, *are1Δ*, *are2Δ*, *tgl1Δ*, *tgl3Δ*, *tgl4Δ* and tgl5Δ mutations on CLS in yeast cultured under CR conditions WT, *dga1Δ*, *are1Δ*, *are2Δ*, *tgl1Δ*, *tgl3Δ*, *tgl4Δ* and *tgl5Δ* cells were cultured in the nutrient-rich YP medium under CR conditions on 0.2% glucose. (**A**, **B**) Survival curves of chronologically aging WT and mutant cells. Data are presented as means ± SEM (*n* = 3). (**C**, **D**) *p* Values for different pairs of survival curves of WT and mutant strains. Survival curves shown in (A, B) were compared. Two survival curves were considered statistically different if the *p* value was less than 0.05. The *p* values for comparing pairs of survival curves using the logrank test were calculated as described in “Materials and Methods”. Abbreviations: L, D, PD and ST, logarithmic, diauxic, post-diauxic and stationary growth phases (respectively).

We found that in CR yeast cultured on 0.2% glucose, the *dga1Δ*, *are1Δ* and *are2Δ* mutations (which eliminate redundant ER enzymes involved in TAG synthesis from FFA and DAG) exhibit the following effects: 1) they increase the concentrations of FFA (Supplementary Figure 5A) and DAG (Supplementary Figure 5B) during L, D and PD phases; 2) they decrease TAG concentration during D and PD phases (Supplementary Figure 5C); 3) they make cells progressing through PD and ST phases more sensitive to liponecrotic RCD (Supplementary Figure 6A); and 4) they significantly shorten yeast CLS (Figure 12A and 12C). We also found that in CR yeast cultured on 0.2% glucose, the *tgl1Δ*, *tgl3Δ*, *tgl4Δ* and *tgl5D* mutations (which eliminate redundant LD lipases catalyzing the degradation of TAG into FFA and DAG) display the following effects: 1) they decrease the concentrations of FFA (Supplementary Figure 5D) and DAG (Supplementary Figure 5E) during L, D and PD phases; 2) they increase TAG concentration during L, D, PD and ST phases (Supplementary Figure 5F); 3) they make cells advancing through PD and ST phases more resistant to liponecrotic RCD (Supplementary Figure 6B); and 4) they significantly extend yeast CLS (Figure 12B and 12D).

In sum, these findings imply that peroxisomal fatty acid β-oxidation makes an essential contribution to yeast CLS extension by CR in part because it decreases the cellular concentrations of FFA and DAG during PD and ST phases [13, 18], thus slowing down the onset of an age-related mode of liponecrotic RCD during these phases of culturing.

## DISCUSSION

**Figure 13:**
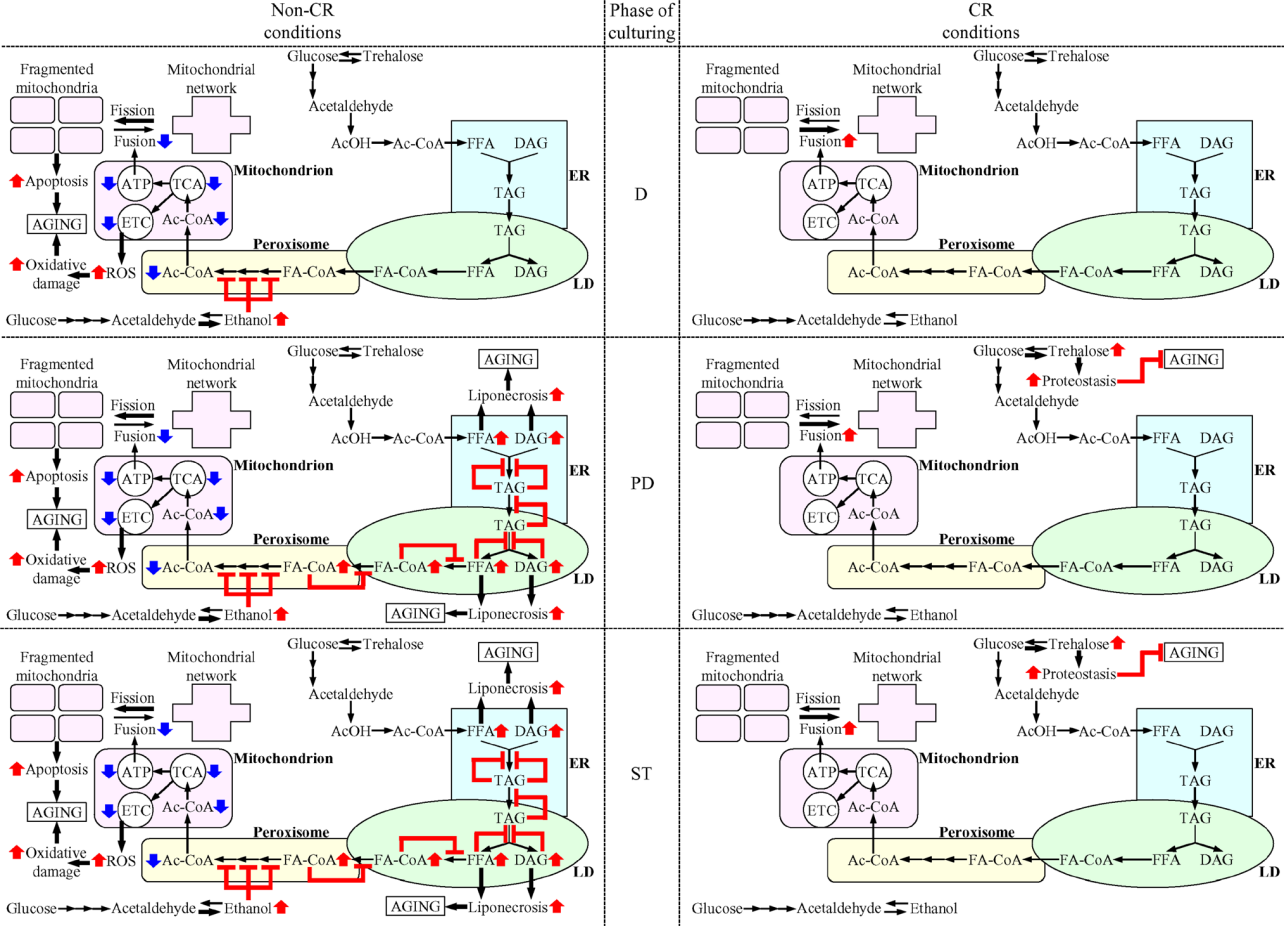
Mechanisms through which CR delays yeast chronological aging by coordinating the spatiotemporal dynamics of various cellular processes CR orchestrates the development and maintenance of distinct patterns of metabolism, interorganellar communications and mitochondrial morphology in yeast cells advancing through D, PD and ST phases of culturing. Throughout different stages of chronological aging, these CR-driven patterns 1) delay the age-related onsets of apoptotic and liponecrotic modes of regulated cell death, thereby decreases the risk of cell death; and 2) preserve cellular proteostasis, thus actively increasing the chance of survival. Because CR decreases the risk of death and actively increases the chance of survival throughout chronological lifespan, this low-calorie diet extends longevity of chronologically aging yeast. The thickness of black arrows is proportional to the rates of processes. Arrows next to the names of affected processes denote those of them that are intensified (red arrows) or weakened (blue arrows). Arrows next to the names of affected metabolites signify those of them whose concentrations are increased (red arrows) or decreased (blue arrows). Inhibition bars are displayed in red color. Please see text for additional details. Abbreviations: Ac-CoA, acetyl-CoA; AcOH, acetic acid; DAG, diacylglycerols; D, PD and ST, diauxic, post-diauxic and stationary growth phases (respectively); ER, endoplasmic reticulum; FA-CoA, acyl-CoA esters; FFA, free fatty acids; LD, lipid droplets; TAG, triacylglycerols; TCA, tricarboxylic cycle.

This study revealed that CR delays yeast chronological aging via mechanisms that coordinate the spatiotemporal dynamics of various cellular processes. Our comparative analyzes of morphological, biochemical and cell biological properties of CR and non-CR yeast advancing through different stages of the aging process suggest a hypothetical model for such mechanisms. This model is depicted schematically in Figure 13.

The model posits that CR orchestrates the development and maintenance of an aging-delaying cellular pattern throughout yeast chronological lifespan, before an arrest of cell growth and division (i.e. during D and PD phases of culturing) and after such arrest (i.e. during ST phase of culturing) (Figure 13). CR elicits the stepwise development and maintenance of this cellular pattern by modulating a network that integrates the following: 1) pathways of ethanol, trehalose and lipid metabolism; 2) interorganellar communications involving unidirectional and bidirectional movements of certain metabolites between the ER and the cytosol, the ER and LD, LD and peroxisomes, and peroxisomes and mitochondria; and 3) a balance between the processes of mitochondrial fission and fusion (Figure 13). These CR-driven patterns of metabolism, interorganellar communications and mitochondrial morphology delay the age-related onsets of apoptotic and liponecrotic modes of RCD throughout different stages of chronological aging (Figure 13). Moreover, CR lowers the concentration of mitochondrially produced ROS and rises trehalose concentration, thereby preserving cellular proteostasis and actively increasing the chance of survival. Because CR decreases the risk of cell death and actively increases the chance of cell survival throughout chronological lifespan (i.e. during D, PD and ST phases of culturing), this low-calorie diet extends yeast CLS.

Specifically, we found that non-CR yeast cells progressing through D phase of culturing accumulate ethanol, a product of glucose fermentation (Figure 13). Such accumulation of ethanol in non-CR yeast during D phase elicits a decline in the concentrations of the core enzymes of peroxisomal fatty acid β-oxidation Fox1, Fox2 and Fox3. The decline of Fox1, Fox2 and Fox3 in non-CR yeast during D phase decelerates peroxisomal fatty acid β-oxidation, thus lowering the pool of peroxisomally produced acetyl-CoA available for mitochondrial oxidation (Figure 13). The ensuing weakening of mitochondrial functionality in non-CR yeast during D phase is manifested as a decline in mitochondrial respiration, *Δ*Ψ, TCA and ATP synthesis, as well as a rise in mitochondrially produced ROS. Because of the decline in mitochondrial functionality and ATP synthesis taking place in non-CR cells during D phase, their mitochondrial network undergoes fragmentation; this initiates the onset of the mitochondria-controlled apoptotic mode of RCD and increases the risk of death (Figure 13). Due to the rise in mitochondrially produced ROS occurring in non-CR cells during D phase, their proteins undergo oxidative damage; this impairs cellular proteostasis and decreases the chance of survival (Figure 13). Owing to the ability of CR to accelerate ethanol consumption and to cause ethanol depletion during D phase, this low-calorie diet 1) preserves mitochondrial respiration, *Δ*Ψ, TCA and ATP synthesis during D phase; 2) prevents mitochondrial network fragmentation during D phase by shifting a balance between the processes of mitochondrial fission and fusion toward fusion; 3) allows to maintain the concentration of mitochondrially produced ROS during D phase below a toxic threshold (Figure 13). This ability of CR to cause ethanol depletion during D phase allows to delay the onset of apoptotic RCD and to maintain cellular proteostasis, thus decreasing the risk of death and actively increasing the chance of survival for cells advancing through D phase of culturing (Figure 13).

Because ethanol concentration remains high in non-CR yeast cells progressing through PD phase of culturing, the risk of death for these cells remains elevated due to the accelerated onset of the mitochondria-controlled apoptotic mode of RCD (Figure 13). In contrast, CR decreases the risk of death during PD phase by allowing to delay the onset of this mode of mitochondria-controlled RCD (Figure 13). Furthermore, the high concentration of ethanol in non-CR yeast cells advancing through PD phase weakens the β-oxidation of FFA in peroxisomes and causes an accumulation of FA-CoA in pexopodia (Figure 13). Such accumulation of FA-CoA in pexopodia of non-CR yeast progressing through PD phase creates several negative feedback loops whose action elicits a build-up of FFA and DAG in the ER and LD (Figure 13). This build-up of FFA and DAG in non-CR yeast accelerates the onset of the liponecrotic mode of RCD during PD phase, thus increasing the risk of death during this phase of culturing (Figure 13). In contrast, CR decreases the risk of death during PD phase by 1) promoting peroxisomal fatty acid β-oxidation; 2) preventing FA-CoA accumulation in pexopodia; 3) allowing to maintain low concentrations of FFA and DAG; and, ultimately 4) delaying the onset of liponecrotic RCD (Figure 13). Moreover, CR actively increases the chance of survival for cells advancing through PD phase of culturing because this low-calorie diet significantly increases the concentration of trehalose, thereby preserving cellular proteostasis (Figure 13).

The risk of death for non-CR yeast cells during ST phase remains high because of the accelerated onsets of both apoptotic and liponecrotic modes of RCD (Figure 13). However, we found that liponecrotic RCD is a prevailing mode of death in non-CR yeast advancing through ST phase (compare Figures 9C and 11). This finding indicates that the apoptotic and liponecrotic modes of RCD may have different relative contributions to the age-related death of non-CR yeast at different periods of CLS. The apoptotic mode of RCD predominates during D phase, apoptotic and liponecrotic RCD modes equally increase the risk of death during PD phase, whereas the liponecrotic mode of RCD prevails during ST phase (Figure 13). This is similar to the ″big P″ and ″small p″ modes of death in the nematode *Caenorhabditis elegans*, which define lifespan earlier or later in life (respectively) [54]. Similar to its effects in yeast progressing through PD phase, CR decreases the risk of death during ST phase by allowing to delay the onsets of both apoptotic and liponecrotic RCD modes (Figure 13). In addition, akin to its effect on the survival of yeast during PD phase, CR increases the chance of survival for yeast progressing through ST phase by rising the concentration of trehalose; this allows CR to maintain cellular proteostasis during ST phase (Figure 13).

In conclusion, this study provides important new insights into mechanisms through which CR delays yeast chronological aging by orchestrating a stepwise remodeling of numerous cellular processes that are integrated into an intricate network. These processes are confined to different cellular locations and occur at different periods of yeast chronological lifespan.

Of note, exogenously added lithocholic bile acid (LCA), a geroprotective chemical compound of mammalian origin, delays yeast chronological aging under CR conditions [39, 48, 55, 56]. In the future, it would be interesting to explore how LCA influences the spatiotemporal dynamics of a network integrating the metabolic pathways and interorganellar communications whose modulation by CR extends longevity of chronologically aging yeast.

## MATERIALS AND METHODS

### Yeast strains, media and growth conditions

The wild-type strain *Saccharomyces cerevisiae* BY4742 (*MAT*α *his3Δ1 leu2Δ0 lys2Δ0 ura3Δ0*) from Open Biosystems/Dharmacon (a part of GE Healthcare) was grown in YP medium (1% yeast extract, 2% peptone; both from Fisher Scientific; #BP1422–2 and #BP1420–2, respectively) initially containing 0.2% (w/v), 0.5% (w/v), 1% (w/v) or 2% (w/v) glucose (#D16–10; Fisher Scientific) as carbon source. Cells were cultured at 30° C with rotational shaking at 200 rpm in Erlenmeyer flasks at a ″flask volume/medium volume″ ratio of 5:1.

### Chronological life span assay

A sample of cells was taken from a culture at a certain time-point. A fraction of the sample was diluted in order to determine the total number of cells using a hemocytometer. Another fraction of the cell sample was diluted and serial dilutions of cells were plated in duplicate onto YP plates containing 2% glucose as carbon source. After 2 d of incubation at 30° C, the number of colony forming units (CFU) per plate was counted. The number of CFU was defined as the number of viable cells in a sample. For each culture, the percentage of viable cells was calculated as follows: (number of viable cells per ml/total number of cells per ml) × 100. The percentage of viable cells in mid-logarithmic phase was set at 100%. The life span curves were validated using a LIVE/DEAD yeast viability kit (Invitrogen) following the manufacturer’s instructions.

### Ethanol concentration measurement

For measuring ethanol and acetic acid concentrations, 1-ml aliquots of yeast cultures were centrifuged and supernatants frozen at −80° C. The supernatants were subjected to gas chromatography using an Agilent 6890 Networked GC system equipped with a Supelco Equity-1 (0.32 mm × 30 cm) column and FID detector. Ethanol and acetic acid concentrations were calculated using the Chemstation 3 software (Agilent).

### Measurement of trehalose and glycogen concentrations

2 × 10^9^ cells were harvested by centrifugation for 1 min at 16,000 × g at 4° C. The cell pellet was washed three times in ice-cold PBS (20 mM KH_2_PO_4_/KOH (pH 7.5) and 150 mM NaCl) and then resuspended in 200 μl of ice-cold SHE solution (50 mM NaOH and 1 mM EDTA). 800 μl of ice-cold SHE solution were added to the cell suspension. The resulting alkali extract was incubated at 60° C for 30 min to destroy endogenous enzyme activities and pyridine nucleotides. The extract was neutralized by adding 500 μl of THA solution (100 mM Tris/HCl (pH 8.1) and 50 mM HCl). The extract was then divided into 150 μl aliquots, quickly frozen in liquid nitrogen and stored at −80° C prior to use.

To measure trehalose concentration, 50 μl of alkali extract were added to 150 μl of trehalose reagent (25 mM KH_2_PO_4_/KOH (pH 7.5) and 0.02% BSA) with or without 15 mU trehalase. The mixture was incubated for 60 min at 37° C. 800 μl of glucose reagent (100 mM Tris/HCl (pH 8.1), 2 mM MgCl_2_, 1 mM DTT, 1 mM ATP, 0.2 mM NADP^+^, and mixture of hexokinase (7 U) and glucose-6-phosphate dehydrogenase (8 U)) were added, and the mixture was incubated for 30 min at 25° C. The NADPH generated from NADP+ was measured fluorimetrically (excitation at 365 nm, emission monitored at 460 nm).

To measure glycogen concentration, 50 μl of alkali extract were added to 500 μl of glycogen reagent (50 mM sodium acetate (pH 4.6) and 0.02% BSA; with and without 5 μg/ml amyloglucosidase 14 U/mg). The mixture was incubated for 30 min at 25° C. 500 μl of glucose reagent (100 mM Tris/HCl (pH 8.1), 2 mM MgCl_2_, 1 mM DTT, 1 mM ATP, 0.2 mM NADP^+^, and mixture of hexokinase (7 U) and glucose-6-phosphate dehydrogenase (8 U)) were added, and the mixture was incubated for 30 min at 25° C. The NADPH generated from NADP+ was measured fluorimetrically (excitation at 365 nm, emission monitored at 460 nm).

### Lipid extraction, separation by thin-layer chromatography (TLC), visualization and quantitation

The recovered pellet of membranes (total cellular membranes, membranes of the purified ER or membranes of purified LD) that contained 1 mg of membrane protein was resuspended in 1.0 ml of chloroform/methanol (1:1; vol/vol). After incubation on ice for 15 min with occasional agitation, the samples were subjected to centrifugation at 20,000 × g for 15 min at 4° C. The chloroform phase was separated and dried under nitrogen. The lipid film was dissolved in 100 μl of chloroform. 25 μl of each sample were spotted on 60-Å silica gel plates for TLC (Whatman). The lipids were developed in the chloroform/acetone (4.6:0.4) [vol/vol] solvent system, detected using 5% phosphomolybdic acid in ethanol and visualized by heating for 30 min at 110° C. The lipids were quantitated by densitometric analysis of TLC plates, using lipid standards in the 0.1–0.5 μg range for calibration.

### Electron microscopy and morphometric analysis

Whole cells were fixed in 1.5% KMnO_4_ for 20 min at room temperature, dehydrated by successive incubations in increasing concentrations of ethanol, and embedded in Poly/Bed 812 epoxy resin (Polysciences). Ultrathin sections were cut using an Ultra-Cut E Microtome (Reichert-Jung). Silver/gold thin sections from the embedded blocks were examined in a transmission electron microscope (JEM-2000FX; JEOL). For morphometric analysis of random electron microscopic sections of cells, 12- × 14-cm prints and 8- × 10-cm negatives of 35–40 cell sections of each strain at 24,000–29,000 magnification were scanned and converted to digitized images with a ScanJet 4400c (Hewlett-Packard) and Photoshop 6.0 software (Adobe Systems, Inc.). Quantitation of digitized images was performed using the Discovery Series Quantity One 1-D Analysis Software (Bio-Rad Laboratories).

### Cellular respiration measurement

A sample of cells was taken from a culture at a certain time-point. Cells were pelleted by centrifugation and resuspended in 1 ml of fresh YP medium containing 0.05% glucose. Oxygen uptake by cells was measured continuously in a 2-ml stirred chamber using a custom-designed biological oxygen monitor (Science Technical Center of Concordia University) equipped with a Clark-type oxygen electrode.

### Fluorescence microscopy

BODIPY 493/503 (Invitrogen) staining for monitoring neutral lipids deposited in lipid droplets and DHR (Sigma) staining for ROS were performed according to established procedures [4]. The mitochondrial membrane potential (∆ψ) was measured in live yeast by fluorescence microscopy of Rhodamine 123 (R123) staining. For R123 staining, 5 × 10^6^ cells were harvested by centrifugation for 1 min at 21,000 × g at room temperature and then resuspended in 100 μl of 50 mM sodium citrate buffer (pH 5.0) containing 2% glucose. R123 (Invitrogen) was added to a final concentration of 10 μM. Following incubation in the dark for 30 min at room temperature, the cells were washed twice in 50 mM sodium citrate buffer (pH 5.0) containing 2% glucose and then analyzed by fluorescence microscopy. Images were collected with a Zeiss Axioplan fluorescence microscope (Zeiss) mounted with a SPOT Insight 2 megapixel color mosaic digital camera (Spot Diagnostic Instruments). For evaluating the percentage of BODIPY 493/503-, DHR- and R123-positive cells, the UTHSCSA Image Tool (Version 3.0) software was used to calculate both the total number of cells and the number of stained cells. Fluorescence of individual DHR- or R123-positive cells in arbitrary units was determined by using the UTHSCSA Image Tool software (Version 3.0). In each of 3 independent experiments, the value of median fluorescence was calculated by analyzing at least 800–1000 cells that were collected at each time-point. The median fluorescence values were plotted as a function of the number of days cells were cultured.

### Immunofluorescence microscopy

Cell cultures were fixed in 3.7% formaldehyde for 45 min at room temperature. The cells were washed in solution B (100 mM KH_2_PO_4_/KOH pH 7.5, 1.2 M sorbitol), treated with Zymolyase 100T (MP Biomedicals, 1 μg Zymolyase 100T/1 mg cells) for 30 min at 30° C and then processed as previously described [57]. Monoclonal antibody raised against porin (Invitrogen, 0.25 μg/μl in TBSB buffer [20 mM Tris/HCl pH 7.5, 150 mM NaCl, 1 mg/ml BSA]) was used as a primary antibody. Alexa Fluor 568 goat anti-mouse IgG (Invitrogen, 2 μg/μl in TBSB buffer) was used as a secondary antibody. The labeled samples were mounted in mounting solution (16.7 mM Tris/HCl pH 9.0, 1.7 mg/ml p-phenylenediamine, 83% glycerol). Images were collected with a Zeiss Axioplan fluorescence microscope (Zeiss) mounted with a SPOT Insight 2 megapixel color mosaic digital camera (Spot Diagnostic Instruments).

### ATP measurement

2 × 10^9^ cells were harvested by centrifugation for 1 min at 16,000 × g at 4° C. The cell pellet was washed three times in ice-cold PBS (20 mM KH_2_PO_4_/KOH (pH 7.5) and 150 mM NaCl) and then resuspended in 200 μl of ice-cold SHE solution (50 mM NaOH and 1 mM EDTA). 800 μl of ice-cold SHE solution were added to the cell suspension. The resulting alkali extract was incubated at 60° C for 30 min to destroy endogenous enzyme activities and pyridine nucleotides. The extract was neutralized by adding 500 μl of THA solution (100 mM Tris/HCl (pH 8.1) and 50 mM HCl). The extract was then divided into 150 μl aliquots, quickly frozen in liquid nitrogen and stored at −80° C prior to use. For ATP measurement, 1 μl of alkali extract was added to 1 μl ATP reagent (50 mM Tris-HCl (pH 8.1), 3 mM MgCl_2_, 0.2 mM glucose, 0.04% BSA, 0.05 mM NADP+, 10 μg/ml hexokinase, 2 μg/ml glucose-6-phosphate dehydrogenase). Following a 30-min incubation at 25° C, the reaction was stopped by adding 1 μl of 0.15 M NaOH and heating at 80° C for 20 min. A 1 μl aliquot was transferred 100 μl of reagent containing 100 μg/ml hexokinase and 20 μg/ml glucose-6-phosphate dehydrogenase. The reaction mixture was incubated for 1 h at 38° C. The NADPH generated from NADP+ was measured fluorimetrically (excitation at 365 nm, emission monitored at 460 nm).

### Total cell lysates preparation

Total cell lysates were made by vortexing the cells in TCL buffer (25 mM Tris/HCl pH 8.5, 150 mM NaCl, 1 mM EDTA, 0.1 mM DTT, 4% CHAPS, 1 mM PMSF, protease inhibitor cocktail [Sigma]) with glass beads three times for 1 min. Lysates were then centrifuged for 3 min at 21,000 × g at 4° C and supernatants collected.

### Cell viability assay for monitoring the susceptibility of yeast to a mode of cell death induced by palmitoleic acid (POA)

A sample of cells was taken from a culture on a certain day of culturing. A fraction of the sample was diluted in order to determine the total number of cells using a hemocytometer. 8 × 10^7^ cells were harvested by centrifugation for 1 min at 21,000 × g at room temperature and resuspended in 8 ml of YP medium containing 0.2% glucose as carbon source. Each cell suspension was divided into 8 equal aliquots. Three pairs of aliquots were supplemented with POA (#P9417; Sigma) from a 50 mM stock solution (in 10% chloroform, 45% hexane and 45% ethanol; #650498, #248878 and #34852, respectively; all from Sigma). The final concentration of POA was 0.05 mM, 0.1 mM or 0.15 mM for each pair of aliquots; in all these aliquots, the final concentrations of chloroform, hexane and ethanol were 0.03%, 0.135% and 0.135%, respectively. One pair of aliquots was supplemented only with chloroform, hexane and ethanol added to the final concentrations of 0.03%, 0.135% and 0.135%, respectively. All aliquots were then incubated for 2 h at 30° C on a Labquake rotator (#400110; Thermolyne/Barnstead International) set for 360° rotation. Serial dilutions of cells were plated in duplicate onto plates containing YP medium with 2% glucose as carbon source. After 2 d of incubation at 30° C, the number of colony forming units (CFU) per plate was counted. The number of CFU was defined as the number of viable cells in a sample. For each aliquot of cells exposed to POA, the % of viable cells was calculated as follows: (number of viable cells per ml in the aliquot exposed to POA/number of viable cells per ml in the control aliquot that was not exposed to POA) × 100.

### Cell viability assay for monitoring the susceptibility of yeast to a mode of cell death induced by hydrogen peroxide

A sample of cells was taken from a culture on a certain day of culturing. A fraction of the sample was diluted in order to determine the total number of cells using a hemocytometer. 8 × 10^7^ cells were harvested by centrifugation for 1 min at 21,000 × g at room temperature and resuspended in 8 ml of YP medium containing 0.2% glucose as carbon source. Each cell suspension was divided into 8 equal aliquots. Three pairs of aliquots were supplemented with hydrogen peroxide (#H325–500; Fisher Scientific) to the final concentration of 0.5 mM, 1.5 mM or 2.5 mM for each pair. One pair of aliquots remained untreated. All aliquots were then incubated for 2 h at 30° C on a Labquake rotator (#400110; Thermolyne/Barnstead International) set for 360° rotation. Serial dilutions of cells were plated in duplicate onto plates containing YP medium with 2% glucose as carbon source. After 2 d of incubation at 30° C, the number of CFU per plate was counted. The number of CFU was defined as the number of viable cells in a sample. For each aliquot of cells exposed to hydrogen peroxide, the % of viable cells was calculated as follows: (number of viable cells per ml in the aliquot exposed to hydrogen peroxide/number of viable cells per ml in the control aliquot that was not exposed to hydrogen peroxide) × 100.

### Statistical analysis

Statistical analysis was performed using Microsoft Excel’s (2010) Analysis ToolPak - VBA. All data on cell survival are presented as mean ± SEM. The *p* values for comparing the means of two groups using an unpaired two-tailed *t* test were calculated with the help of the GraphPad Prism 7 statistics software. The logrank test for comparing each pair of survival curves was performed with GraphPad Prism 7. Two survival curves were considered statistically different if the *p* value was less than 0.05.

### Miscellaneous procedures

Enzymatic activity of cytochrome *c* oxidase measurement [57], a measurement of succinate dehydrogenase enzymatic activity [58], enzymatic activity of aconitase measurement [59], SDS-PAGE and immunoblotting [60], subcellular fractionation of yeast [61], purification of LD [62], purification of the ER [61] were performed as previously described.

## SUPPLEMENTARY MATERIALS FIGURES AND TABLES



## Abbreviations

Ac-CoA: acetyl-CoA
ACO: aconitase
AcOH: acetic acid
CCO: cytochrome *c* oxidase
CLS: chronological lifespan
CR: caloric restriction
D: diauxic growth phase
DAG: diacylglycerols
EE: ergosteryl esters
ER: the endoplasmic reticulum
ETC: the electron transport chain
FA-CoA: acyl-CoA esters
FFA: free fatty acids
IMM: inner mitochondrial membrane
L: logarithmic growth phase
LD: lipid droplets
PD: post-diauxic growth phase
RCD: regulated cell death
ROS: reactive oxygen species
SDH: succinate dehydrogenase
ST: stationary growth phase
TAG: triacylglycerols
WT: wild type.
