# Supplementary material for: Caloric restriction delays yeast chronological aging by remodeling carbohydrate and lipid metabolism, altering peroxisomal and mitochondrial functionalities, and postponing the onsets of apoptotic and liponecrotic modes of regulated cell death
Source: Oncotarget. 2018 Mar 5;9(22):16163–84. doi: 10.18632/oncotarget.24604 (PMC5882325; doi:10.18632/oncotarget.24604)
Supplement: Supplementary file 1 [file oncotarget-09-16163-s001.pdf]

Caloric restriction delays yeast chronological aging by remodeling carbohydrate and lipid metabolism, altering peroxisomal and mitochondrial functionalities, and postponing the onsets of apoptotic and liponecrotic modes of regulated cell death

SUPPLEMENTARY MATERIALS

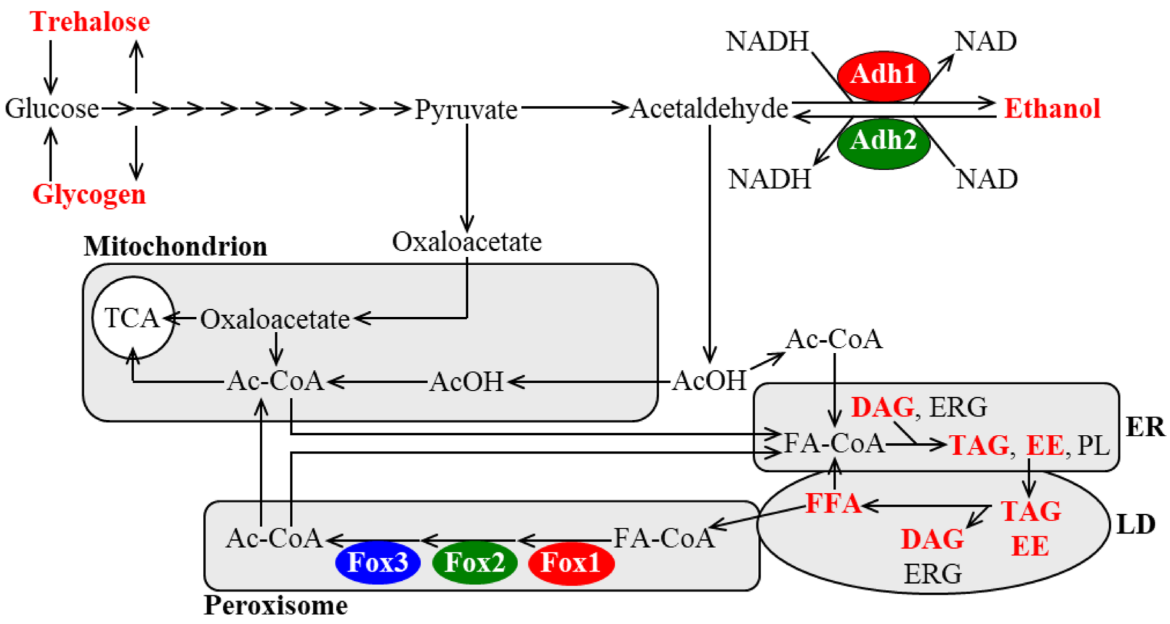

Supplementary Figure 1: The Adh1- and Adh2-dependent metabolism of ethanol is integrated into a network which also assimilates the synthesis and degradation of glycogen and trehalose, the synthesis of neutral lipids TAG and EE in the ER, the lipolytic degradation of TAG and EE in LD, and the Fox1-, Fox2- and Fox3-dependent β-oxidation of FFA in peroxisomes. The names of metabolites integrated into this network and pertinent to this study are displayed in red color. The Adh1 isozyme of alcohol dehydrogenase catalyzes the conversion of acetaldehyde to ethanol, whereas the Adh2 isozyme of alcohol dehydrogenase is involved in a reverse process of ethanol conversion to acetaldehyde. Abbreviations: Ac-CoA, acetyl-CoA; AcOH, acetic acid; DAG, diacylglycerols; EE, ergosteryl esters; ER, endoplasmic reticulum; ERG, ergosterol; FFA, free fatty acids; FA-CoA, fatty acyl-CoA esters; LD, lipid droplets; TAG, triacylglycerols.

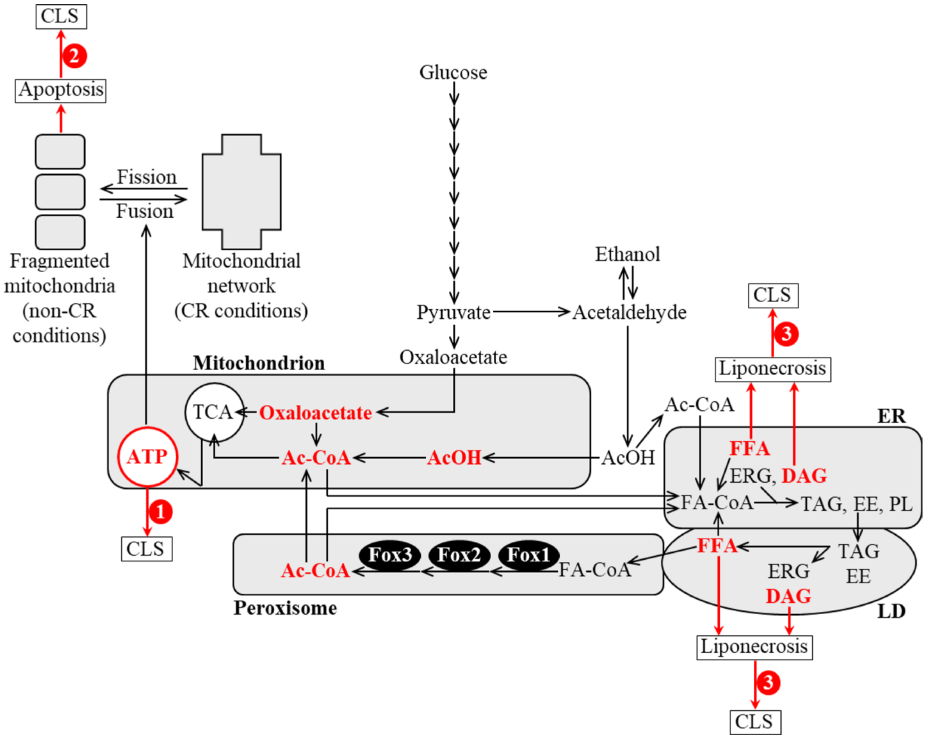

Supplementary Figure 2: Possible mechanisms through which the β-oxidation of FFA in peroxisomes may define longevity of chronologically aging yeast. The names of metabolites whose concentrations depend on the extent of peroxisomal fatty acid β-oxidation are displayed in red color. There may be at least three different mechanisms through which the concentrations of these metabolites may define yeast chronological lifespan (CLS). These mechanisms are numbered. See text for more details. Abbreviations: Ac-CoA, acetyl-CoA; AcOH, acetic acid; CR, caloric restriction; DAG, diacylglycerols; EE, ergosteryl esters; ER, endoplasmic reticulum; ERG, ergosterol; FFA, free fatty acids; FA-CoA, fatty acyl-CoA esters; LD, lipid droplets; TAG, triacylglycerols.

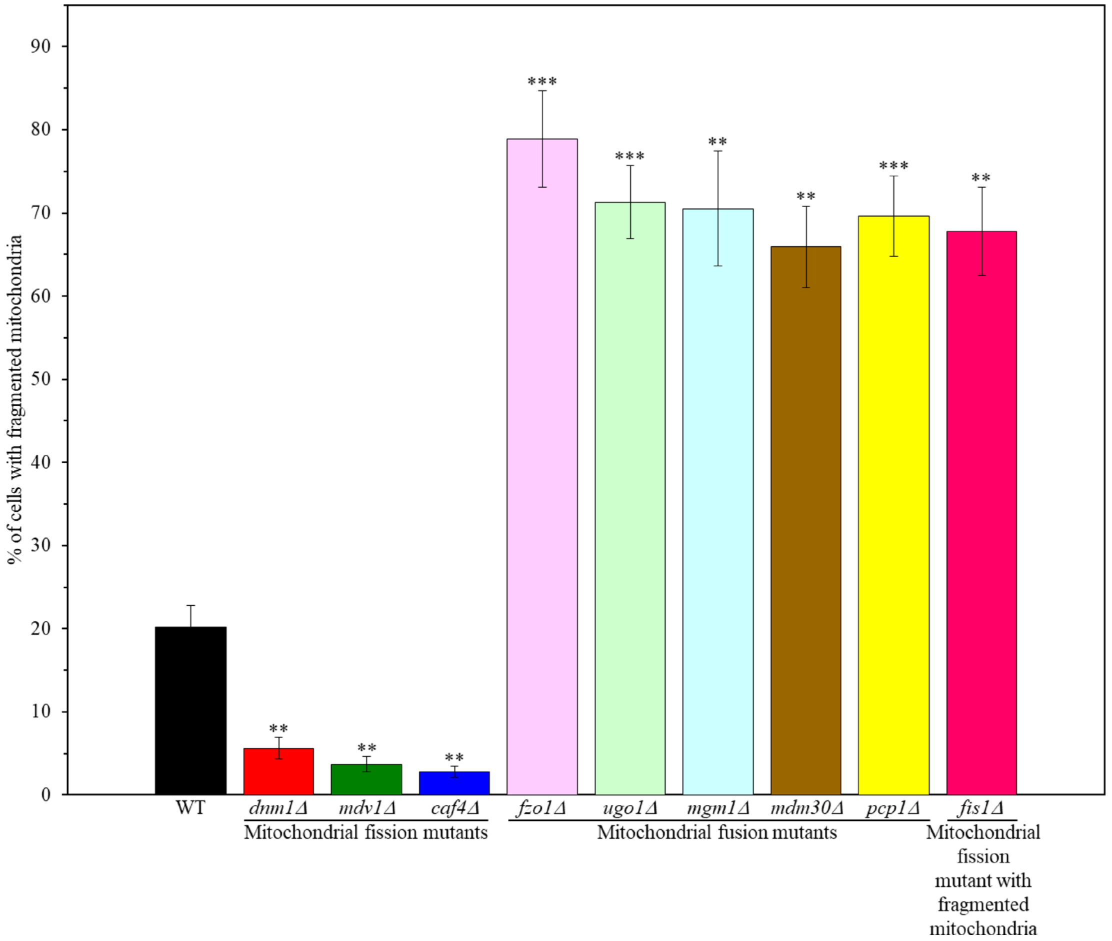

Supplementary Figure 3: Effects of the single-gene-deletion mutations eliminating different protein components of the mitochondrial fission or fusion machine on mitochondrial morphology in yeast cultured under CR conditions. WT, *dnm1Δ*, *mdv1Δ*, *caf4Δ*, *fzo1Δ*, *ugo1Δ*, *mgm1Δ*, *mdm30Δ*, *pcp1Δ* and *fis1Δ* cells were cultured in the nutrient-rich YP medium under CR conditions on 0.2% glucose. Mitochondrial morphology was visualized with the help of indirect immunofluorescence microscopy using primary antibodies against porin, as described in Materials and Methods and shown in Figure 10A. The percentage of cells exhibiting fragmented mitochondria was calculated. At least 500 cells of each strain were used for quantitation. Data are presented as means ± SEM ($n = 3$; $^{**} < 0.01$; $^{***} < 0.001$).

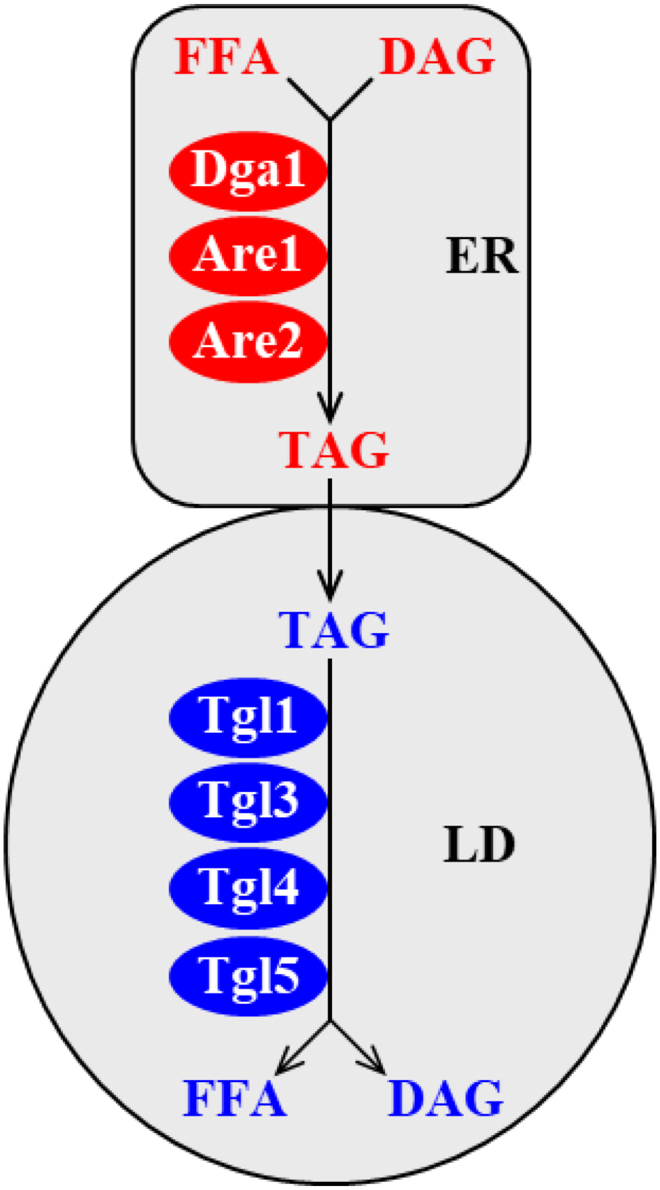

Supplementary Figure 4: TAG synthesis from FFA and DAG in the ER and TAG lipolysis into FFA and DAG in LD are catalyzed by redundant enzymes. See text for more details. Abbreviations: DAG, diacylglycerols; ER, endoplasmic reticulum; FFA, free fatty acids; LD, lipid droplets; TAG, triacylglycerols.

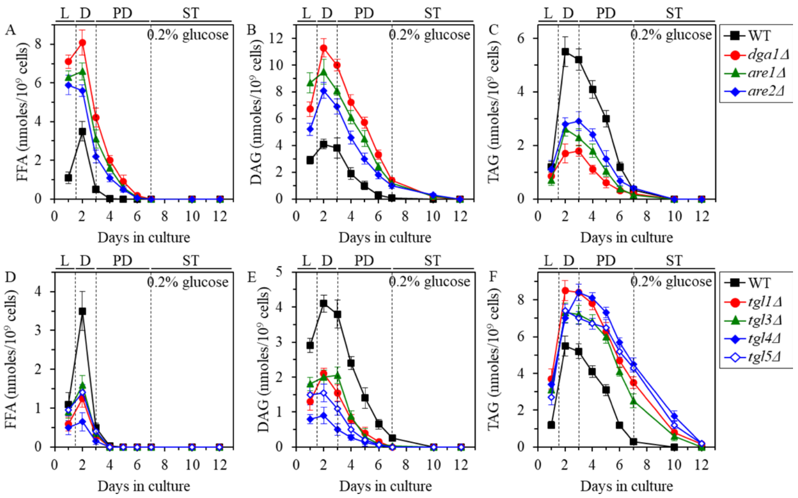

Supplementary Figure 5: Effects of the *dga1Δ*, *are1Δ*, *are2Δ*, *tgl1Δ*, *tgl3Δ*, *tgl4Δ* and *tgl5Δ* mutations on FFA, DAG and TAG concentrations in yeast cultured under CR conditions. WT, *dga1Δ*, *are1Δ*, *are2Δ*, *tgl1Δ*, *tgl3Δ*, *tgl4Δ* and *tgl5Δ* cells were cultured in the nutrient-rich YP medium under CR conditions on 0.2% glucose. FFA (**A** and **D**), DAG (**B** and **E**) and TAG (**C** and **F**) concentrations in whole cells recovered on different days of culturing are shown. Data are presented as means ± SEM (*n* = 3). The concentrations of FFA, DAG and TAG in whole cells were measured as described in Materials and Methods. Abbreviations: DAG, diacylglycerols; FFA, free fatty acids; L, D, PD and ST, logarithmic, diauxic, post-diauxic and stationary growth phases (respectively); TAG, triacylglycerols.

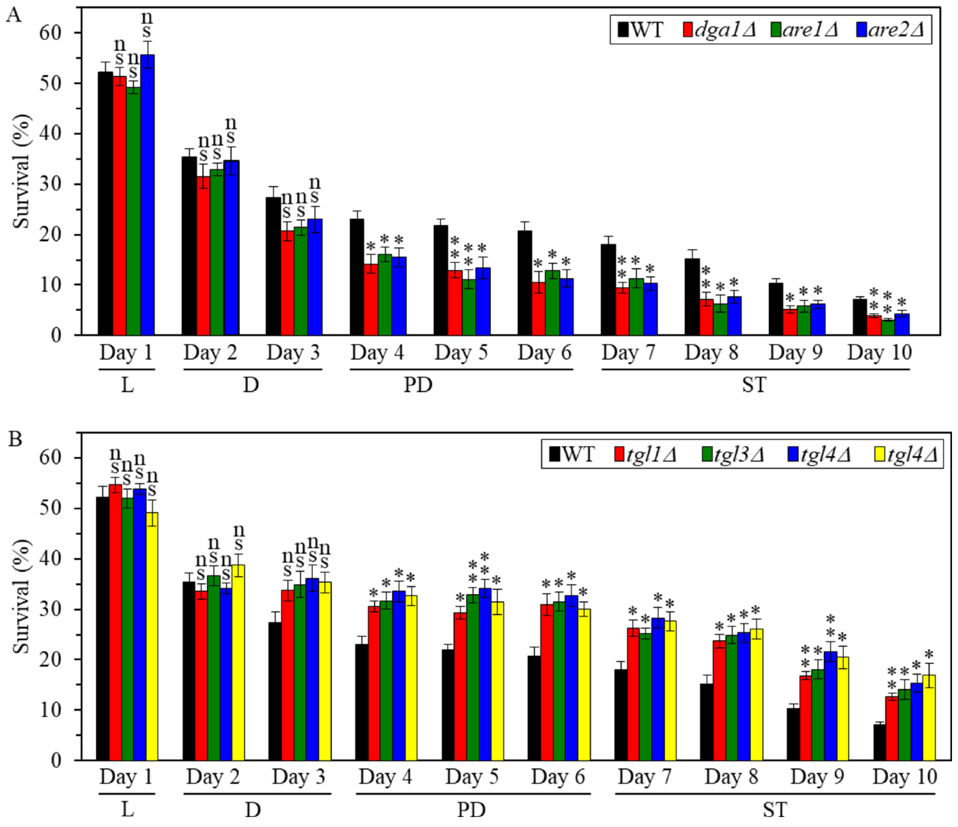

Supplementary Figure 6: Effects of the *dga1Δ*, *are1Δ*, *are2Δ*, *tgl1Δ*, *tgl3Δ*, *tgl4Δ* and *tgl5Δ* mutations on cell susceptibility to liponecrotic RCD in yeast cultured under CR conditions. WT, *dga1Δ*, *are1Δ*, *are2Δ*, *tgl1Δ*, *tgl3Δ*, *tgl4Δ* and *tgl5Δ* cells were cultured in the nutrient-rich YP medium under CR conditions on 0.2% glucose. An assay for measuring clonogenic survival of cells treated for 2 h with 0.15 mM palmitoleic acid, a monounsaturated FFA, was performed as described in Materials and Methods. Data are presented as means ± SEM (*n* = 3; ns, not significant; * < 0.05; ** < 0.01). Abbreviations: L, D, PD and ST, logarithmic, diauxic, post-diauxic and stationary growth phases (respectively).
